# Structuring Disorder via Supervised Molecular Dynamics: Uncovering Arginine-Glycine-Glycine-Mediated Ribonucleic Acid-Intrinsically Disordered Region Recognition Mechanisms

**DOI:** 10.1021/acs.jcim.5c03135

**Published:** 2026-05-07

**Authors:** Gianluca Novello, Andrea Dodaro, Chiara Cavastracci Strascia, Silvia Menin, Mattia Sturlese, Veronica Salmaso, Stefano Moro

**Affiliations:** Molecular Modeling Section (MMS), Department of Pharmaceutical and Pharmacological Sciences, University of Padova, via Marzolo 5, 35131 Padova, Italy

## Abstract

In recent years, RNA has emerged as a central player in gene regulation and cellular homeostasis, far beyond its canonical role as a mediator between DNA and proteins. Moreover, RNA-binding proteins orchestrate many of these processes not only through their folded domains but also via intrinsically disordered regions (IDRs). Particular attention has been given to arginine–glycine-rich motifs, which endow these regions with remarkable versatility, flexibility, and interaction adaptability. However, the dynamic nature of such regions represents a major challenge for both structural characterization and computational modeling of their interactions with RNA. In this study, we explore the applicability of supervised molecular dynamics (SuMD) to reconstruct, at atomic resolution, the recognition mechanisms between RNA and disordered protein regions while capturing the multistep nature of the binding process. By focusing on two experimentally resolved systems, SF3A1-UBL/U1-SL4 and FUS RRM/U1-SL3, we show that SuMD can reproduce association pathways involving both disordered and structured regions, capturing transient contacts and interaction hierarchies. We further extend the approach to a prospective system lacking an experimentally resolved complex structure, leading to a model that is consistent with experimental mutagenesis data. This approach provides new perspectives for understanding how IDRs recognize and modulate RNA and generating structural hypotheses for such complexes, paving the way for future applications in the rational design of RNA–protein-targeted therapeutics

## Graphical abstract

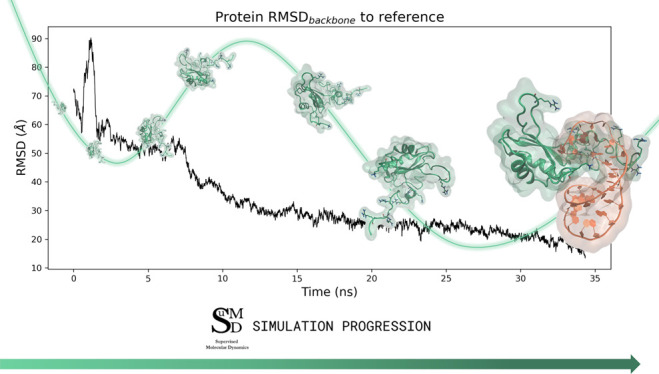

## Introduction

Ribonucleic acid (RNA) plays roles far beyond its canonical function as an intermediary between DNA and proteins, participating in gene regulation, catalyzing biochemical reactions, and maintaining genomic integrity.^1^ While only a small fraction of the transcriptome encodes for proteins, a substantial portion generates noncoding RNAs (ncRNAs), whose dysregulation is associated with numerous diseases, including cancer and neurodegenerative disorders.^2,3^

A growing interest in developing RNA-based therapeutics, aimed at regulating RNA function or gene expression for disease treatment, has emerged alongside recent advances in RNA biology.^4^ In this context, it is pivotal to understand how RNA-binding proteins (RBPs) interact with RNA, forming ribonucleoprotein complexes that are involved in a wide range of cellular processes, including transcriptional and post-transcriptional regulation, as well as the formation of membraneless organelles such as stress granules and nucleoli.^5–7^ While early structural studies primarily focused on folded RNA-binding domains, many RBPs are now known to contain extensive intrinsically disordered regions (IDRs).^8^ These regions lack a stable tertiary structure and interact with RNA through dynamic, multiple contacts, challenging the classical structure–function paradigm.^9^ Among the most prominent motifs found in IDRs are arginine-glycine (RG) and arginine-glycine-glycine (RGG) repeats. Together, RG/RGG domains represent the second most prevalent RNA-binding domains in the human proteome, occurring in over 1,800 proteins with at least two closely spaced repeats.^10,11^ Due to their distinctive molecular features, proteins containing the RGG motif participate in a wide range of cellular processes, including DNA repair, transcription, RNA splicing, and translation.^12^ The planar, positively charged guanidinium group of arginine mediates electrostatic interactions with the negatively charged RNA backbone, as well as hydrogen bonding and cation-π interactions, while adjacent glycine residues confer high backbone flexibility.^12–14^ Together, these attributes confer both specificity and adaptability, allowing RGG-containing proteins to interact with diverse partners and RNA targets.^11^ Despite their biological relevance, high-resolution structures of RG/RGG-RNA complexes remain scarce. To date, only a handful of Protein Data Bank (PDB) entries^15–18^ capture direct contacts between human RG/RGG motifs and RNA, reflecting the experimental challenges associated with these highly flexible and disordered regions. Further structural characterization is critical to understand how these motifs contribute to RNA processing and transport and how altered interactions may compromise physiological functions and promote disease development. From this perspective, computational modeling can serve as a critical tool to investigate and elucidate the molecular mechanisms involved in these processes.

A variety of in silico methods, ranging from molecular docking and molecular dynamics (MD) simulations to recent artificial intelligence (AI)-based structure prediction techniques, have been applied to model RNA–protein complexes, but with still limitations when dealing with IDRs.^19^ AI-based predictors show considerable potential for biomolecular modeling,^20,21^ yet they face inherent limitations in modeling intrinsically disordered regions (IDRs) and protein–RNA complexes,^22,23^ likely due to the shortage of RNA and RNA–protein complexes structures in the training sets.^24^ In particular, AI-based predictors often model IDRs as low-confidence static coils, overlooking both their dynamic association modes and sequence-specific contacts.^24^ On the other hand, molecular docking remains one of the most widely used computational strategies to simulate molecular recognition.^25,26^ Originally developed to study small molecule–protein interactions,^27^ docking methodologies have since been extended to protein–protein^28^ and protein–peptide^29^ systems, and more recently to nucleic acid–protein complexes.^30,31^ While standard docking tools have evolved to handle structured proteins and RNAs with increasing accuracy, their application to disordered protein regions remains difficult. Indeed, the intrinsic flexibility of RNA and many RNA-binding proteins remains a significant obstacle for existing docking frameworks.^32^ The presence of intrinsically disordered proteins (IDPs) or regions (IDRs), which lack a stable tertiary structure and engage in dynamic, multivalent interactions,^33^ further complicates the scenario, making the accurate prediction of these interactions a significant unresolved problem. Integrating molecular dynamics (MD) or other MD-based techniques, either before or after docking, is indispensable for sampling realistic IDR-RNA conformers.^34^ In fact, MD provides a more detailed view of the binding process, explicitly simulating solvent and ions and accounting for the flexibility of both the receptor and the ligand. Over the past few years, both classical all-atom molecular dynamics simulations^35,36^ and coarse-grained MD^37^ have been employed to study RG/RGG and other IDRs-RNA interactions. Although these studies demonstrate the utility of MD for investigating these complexes, they share a critical limitation: simulations are often started from preformed bound states, neglecting the role of disordered regions in the initial target recognition process. Moreover, the high computational cost required to capture such rare binding events typically confines MD to refining docking poses rather than simulating the entire binding pathway.^38^

To overcome these limitations, a variety of enhanced-sampling techniques have been developed to accelerate the exploration of biomolecular recognition processes.^39^ Many approaches, such as metadynamics, umbrella sampling, and steered molecular dynamics, rely on the application of an energetic bias along selected collective variables to enhance sampling.^40^ Other strategies, including replica exchange and Gaussian accelerated MD, aim to improve conformational exploration without requiring predefined reaction coordinates.^41,42^ While these methods have proven highly valuable for characterizing conformational landscapes and binding energetics, their application to RNA-IDR systems remains challenging, considering the risk of mapping nonphysical states and the difficulty of defining collective variables and reweighting energy surfaces. To address these computational constraints, techniques such as supervised molecular dynamics (SuMD) represent an effective alternative. This approach enables the accelerated simulation of ligand–receptor recognition events by running short subsequent classical MD simulations moving forward from states where ligand–receptor (supervised) distance decreases without introducing energy bias to the system. SuMD thereby allows for the efficient reconstruction of binding mechanisms at atomic resolution on time scales that are significantly shorter than those accessible by conventional MD,^43^ and captures the intrinsic flexibility of the entire system, including diverse ligand types (small molecules,^44–49^ macrocycles,^50^ and peptides^51^) and targets (soluble proteins,^52,53^ membrane receptors,^54–56^ and nucleic acids^57–59^ ), making it particularly well suited for investigating RNA binding by IDPs. Unlike enhanced-sampling approaches, SuMD does not modify the underlying potential energy surface but instead adapts sampling by running short unbiased simulations, which are elongated if the ligand–receptor distance shortens, otherwise stopped and repeated.

Leveraging the versatility of SuMD, we investigated its applicability to the recognition of RNA and arginine-glycine-rich (RGG) motifs by adapting the simulation protocol. We examined two experimentally resolved complexes, the ubiquitin-like (UBL) domain of SF3A1 bound to U1 snRNA stem-loop 4 (SL4),^15^ and the RNA recognition motif (RRM) of FUS bound to U1 snRNA stem-loop 3 (SL3).^16^ Specifically, we focused on complexes in which RGG motifs are adjacent to structured domains rather than on fully disordered RGG-only systems, aiming for the characterization of the RGG motifs’ contributions to RNA recognition in a defined structural context. In addition, once we assessed the capability of SuMD to reproduce experimental structures, we prospectively investigated a system for which no high-resolution complex structure is available, involving the intrinsically disordered protein SERF2 and a telomeric G-quadruplex RNA (TERRA12). This made it possible to test whether SuMD could generate a structural hypothesis of RNA-IDP complexes in agreement with interaction experimental data, thus paving the way to the application of SuMD to predictive scenarios.

## Materials and Methods

### Hardware Overview

Structure preparation, setup for the MD simulations, and consequent analysis were performed on a 24-core Intel Core i9–14900K 6.0 GHz processor Linux workstation. Molecular dynamics simulations were performed on a cluster composed of 30 NVIDIA GPUs (GTX 980- RTX4090).

### Structure Preparation

The experimentally determined three-dimensional coordinates of the RNA–protein complexes were retrieved from the Protein Data Bank^60^ (PDB ID: 7P0V,^15^ 6SNJ,^16^ 2KBP,^61^ and 9DT0 ^62^). For the NMR structures (6SNJ, 2KBP, and 9DT0), the first conformer was selected according to the deposited selection criteria. The structure was prepared exploiting several tools from the Molecular Operating Environment (MOE) 2024.06.^63^ Specifically, the ″Structure Preparation″ tool was used to identify and correct the discrepancies present in the initial structure. After removing water molecules and nonprotein or non-nucleic residues, the protein was moved away from the binding site at a distance of at least 30 Å from the nearest receptor atom. The ″Protonate3D″ tool was then applied to replace missing hydrogens in accordance with the protomeric and tautomeric states (pH 7.4, T 310 K, i.f. = 0.154). Subsequently, the AMBER14:ETH force field was employed to assign partial charges and minimize the energy of the hydrogen atoms.

### System Setup and Equilibration protocol for MD Simulations

The RNA–protein complexes for molecular dynamics (MD) simulations were prepared using Visual Molecular Dynamics (VMD)^64^ 1.9.3 and AmberTools22.^65^ The ff14SB force field was used to parametrize each protein or nucleic acid atom, with the χ modification modified for RNA (χ_OL3_).^66–68^ Systems were solvated in a cubic box with a padding of 20 Å between the complex and the box boundary, using the TIP3P water model.^69^ To neutralize the system and reach a physiological salt concentration of 0.154 M, sodium (Na^+^) and chloride (Cl^–^) ions were added. Finally, a 500-step energy minimization process was performed, exploiting the conjugate gradient technique to eliminate clashes and unfavorable contacts.

Before the production phase was started, a two-step equilibration process was used. In the first step, each protein and RNA atom was subjected to a 5 kcal mol^–1^ Å^–2^ harmonic positional constraint during a 1 ns simulation in the canonical ensemble (NVT). In the second equilibration step, the same restraint was exclusively applied to nucleic and protein backbones during an equal-length simulation in the isothermal–isobaric ensemble (NPT). A Monte Carlo barostat was employed to maintain the pressure at 1 atm during the NPT simulation phase,^70^ and the temperature in all the equilibration phases was regulated at 310 K with a Langevin thermostat.^71^

The ACEMD 3.7.6 ^72^ engine, which is based on the open-source Python package OpenMM,^73^ was used for all MD simulations. An integration time step of 2 fs was adopted, and bonds involving hydrogen atoms were constrained using the M-SHAKE method.^74^ Long-range electrostatic interactions were computed using the particle-mesh Ewald approach,^75^ with a switching distance of 7.5 Å for Lennard-Jones interactions and a cutoff of 9.0 Å for Lennard–Jones and real-space electrostatic interactions.

### Supervised Molecular Dynamics (SuMD) Simulation

Supervised molecular dynamics (SuMD) is an established enhanced MD technique employed to study molecular recognition mechanisms with atomic resolution on nanosecond time scales.^43^ Unlike conventional molecular dynamics, which may require microseconds to capture rare events, such as ligand binding, SuMD accelerates the observation of such processes by selectively retaining the simulation steps in which the ligand approaches the target.

The method operates through a series of short, unbiased MD simulations, termed SuMD steps, conducted under the canonical ensemble (NVT) at 310 K, lasting 300 ps. At the conclusion of each step, the distance between the centers of mass of the ligand and a user-defined binding region is calculated at every frame. A linear function is then fitted to these time-dependent distances, and the slope of the line is calculated. If the slope is negative, indicating that the ligand is approaching the binding site, then the step is considered productive and retained. Conversely, if the ligand is not approaching the binding site, the slope is positive, and the step is discarded. The final coordinates of a productive step are used as the starting point for the subsequent one, with randomized velocities generated via the Langevin thermostat. In the current implementation, the SuMD code is written in Python and exploits Numpy and ProDy^76^ modules to carry out the geometrical supervision throughout the simulation. In the specific application developed for this study, the entire trajectory is partitioned into two different supervision steps. Initially, the supervision targets residues located in the disordered region of the protein. Once this disordered segment approaches and stabilizes in contact with the RNA moiety, the supervision is switched to the key residues positioned within the folded region of the protein.

### RNA–Protein Complexes Classical Molecular Dynamics Simulations

Several classical molecular dynamics (MD) simulations were conducted to investigate the conformational dynamics of the selected RNA–protein complexes. Each system was prepared by using AmberTools22 and VMD 1.9.2, following the same equilibration procedure previously described for the SuMD simulations. Subsequently, the reference complexes and the final frames obtained from the corresponding SuMD trajectories were subjected to 100 or 500 ns of classical MD simulations.

### SuMD and Classical MD Trajectory Analysis

The analysis of SuMD and classical MD trajectories was performed using a custom Python 3 tool that extends the capabilities of the original software described by Salmaso et al.,^51^ and allows both structural and energetic properties to be analyzed across the trajectories. Structural geometry across the simulations was evaluated by generating RMSD plots and SuMD center-of-mass distance profiles using the MDAnalysis Python library^77,78^ and Matplotlib.^79^ Energetic profiling involved estimating ligand–receptor interaction energies throughout the trajectory using the NAMD Energy plugin, based on the NAMD engine^80^ and the AMBER14 force field. Interaction energies were calculated as the sum of the van der Waals and electrostatic contributions. Additionally, the per-residue decomposition of the interaction energy, generated through the same plugin, adds the time-resolved energy contributions from protein amino acids and nucleotides of the nucleic acid ligand. Only the 25 most frequently contacting residues from each component were considered, with contacts defined as atom pairs within a maximum distance of 4.5 Å. In addition to trajectory analyses, the Python tool, exploiting VMD, also generated video representations of the trajectories featuring dynamic overlays of structural and energetic metrics. For consistency, all residue numbering shown in plots and videos follows the canonical UniProt^81^ FASTA sequence of the respective receptor (Isoform 2 of Splicing factor 3A subunit 1 Q15459, RNA-binding protein FUS P35637, Small EDRK-rich Factor 2 SERF2, UniProt P84101). The sequences of all constructs used in this study are reported in Table S1 in the Supporting Information.

## Results

The applicability of Supervised Molecular Dynamics (SuMD) in modeling recognition processes between nucleic acids and intrinsically disordered regions (IDRs) was assessed by testing the protocol’s ability to reproduce experimentally determined binding modes between arginine-glycine-rich IDRs-containing proteins and RNAs. Additionally, the method was applied prospectively to a case whose structure has not yet been experimentally resolved: a disordered, non-RGG-containing protein that interacts with RNA through positively charged residues. For the two retrospective cases, starting from experimentally resolved protein–RNA complexes, the protein chain was moved away from the RNA macromolecule, and SuMD was thus used to simulate the full binding pathway. For the third prospective case, the single PDB structures of the protein and RNA were randomly positioned under the same conditions at a distance of 30 Å. In the classical SuMD workflow, previously published,^43,57,58^ short consecutive simulations (SuMD steps) are run, and the distance between the center of mass of the two interacting partners is monitored. At the end of each SuMD step, the collected distances are fitted by a line whose slope is used as a decision-making criterion. If the slope is negative, the simulation proceeds into the next SuMD step; otherwise, the current step is resimulated by reassigning atom velocities. A full trajectory is finally obtained by merging the outcomes of consecutive SuMD steps. It is worth noting that the SuMD code contemplates user-defined indication of the interacting molecules, thus also allowing the selection of molecule substructures for distance monitoring. This is useful when dealing with large and flexible macromolecular ligands, where the center of mass of the molecule does not coincide with the center of mass of the portion involved in target recognition. This applies in the context of IDRs-containing proteins and RNA, where portions of the IDR and the structured domain of the protein can interact with different regions of the RNA counterpart.

To assess the capability of SuMD to reproduce the recognition of RNA and IDR-containing proteins, SuMD was initially applied through the classic workflow of typically monitoring the distance between the centers of mass of the whole RNA and the whole IDR-containing protein. These attempts turned out to be unproductive (data not shown).

Thus, in this work, a two-phase supervision protocol was introduced, referred to as Supervision Step-1 and Step-2, comprising a monitoring of the distance between RNA and the unstructured protein region first and the structured region second. More precisely, during Step-1, the approach of the intrinsically disordered region of the protein toward the RNA was monitored, whereas Step-2 focused on the supervision of key residues located within the more structured regions of the protein. The order of supervision was based on preliminary simulations where the first RNA–protein contacts were observed to engage the protein unstructured region, as described in detail below. For each test case, three independent replicates starting from the same initial configuration of the system were conducted.

Two experimentally resolved complexes were used as retrospective validation systems, with the aim to assess the capability of the two-step SuMD approach to reproduce the complex experimental structure: the ubiquitin-like (UBL) domain of SF3A1 bound to U1 snRNA stem-loop 4 (SL4) (PDB 7P0V, X-ray) and the RNA recognition motif (RRM) of FUS bound to U1 snRNA stem-loop 3 (SL3) (PDB ID: 6SNJ, NMR). As a prospective test case, we investigated the interaction between SERF2 (Small EDRK-rich Factor 2; PDB ID: 9DT0, NMR) and the G-quadruplex RNA TERRA12 (PDB ID: 2KBP, NMR), for which individual structures are available, but no experimentally resolved complex has been reported.

For the retrospective systems, the performance of SuMD was evaluated by assessing the ability of the method to reproduce the experimentally determined binding geometries together with the corresponding interaction energy fingerprints, thereby evaluating not only structural agreement but also the preservation of the interaction network. In the prospective case, the same analyses were applied to characterize the interaction profile of the predicted binding mode and its agreement with experimental per-residue binding data (NMR-HSQC data), in the absence of a structural reference.

The three case studies are presented below with an increasing order of structural complexity. The first involves a protein containing a single RGG repeat bound to a relatively small RNA segment. The second feature a protein with multiple RGG repeats, a structured RRM domain, and a larger RNA stem-loop. The third case examines the interaction between the intrinsically disordered non-RGG protein SERF2 and the structured G-quadruplex RNA TERRA12.

### U1 snRNA Stem-Loop 4 in Complex with Human SF3A1 Ubiquitin-Like Domain (PDB ID: 7P0V)

The human SF3A1 protein is a subunit of the heterotrimeric SF3A complex, which, together with the SF3B complex, plays a pivotal role in the early stages of the splicing process.^82,83^ This initial interaction involves the direct binding of SF3A1, specifically through its UBL (ubiquitin-like) domain, to the stem-loop 4 (SL4) region of U1 snRNA.^84^ In 2022, the first high-resolution structure of this complex was resolved, highlighting the critical contribution of the C-terminal disordered region of SF3A1’s UBL domain, characterized by a positively charged RGGR motif.^15^ The resolved structure includes the UBL domain (residues 704–791), comprising a globular β-sheet-rich core and an unfolded C-terminal tail bearing the RGGR motif. This C-terminal segment inserts into the major groove of the U1-SL4 RNA (residues 139–162), establishing shape- and sequence-specific interactions together with the globular portion of the UBL domain. Key residues involved in RNA recognition include Arg788 and Arg791, within the C-terminal tail, which establish strong electrostatic and hydrogen bonding interactions with the RNA. Gly789 and Gly790, also part of the same region, contribute by enhancing local flexibility and facilitating insertion into the RNA groove. Additionally, several residues from the globular domain, i.e., Lys717, Lys754, Lys756, Lys765, and Lys786, interact with RNA, complementing the role of the disordered tail.

To preliminarily evaluate the stability of the deposited complex and characterize the interaction network in the native conformation, a 500 ns classical molecular dynamics simulation was performed. The results of this analysis are shown in Figure 1. During the simulation, both the RNA and protein components of the complex remained mostly stable, as shown by the backbone RMSD profiles of the protein and RNA (Figure 1A,B). Relevant insight emerges from the per-residue interaction energy profiles reported in Figure 1C,D. All the protein residues previously identified as key for complex formation display favorable and stable energetic contributions throughout the simulation (Figure 1D), confirming their central role in mediating binding to the most contacted nucleotides like G140, G141, C144, U145, G146, G148, U149, U150, C151, G154, and C155 (Figure 1C).

**Figure 1:**
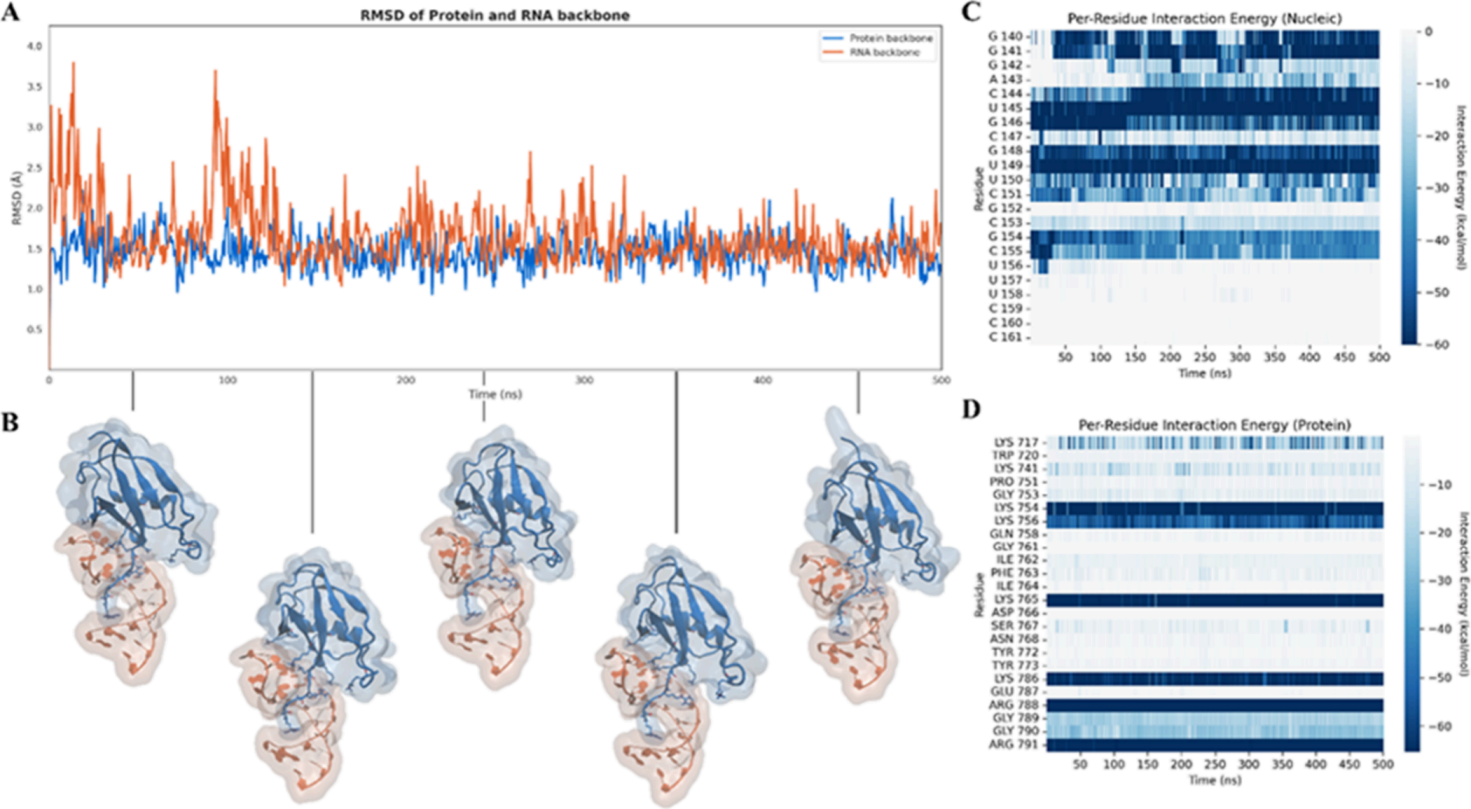
(A) Root-mean-square deviation (RMSD) of the protein SF3A1 (blue) and RNA U1-SL4 (orange) backbones during the molecular dynamics simulation, indicating the overall structural stability of the complex. (B) Representative conformations extracted at selected time points, illustrating the relative orientation and conformational stability of the protein (blue) and RNA (orange) throughout the trajectory. (C, D) Per-residue interaction energy profiles for the nucleic acid (C) and protein (D) components computed over the course of the simulation, highlighting the key residues contributing to complex stability in the crystallographic model.

Based on these analyses, key protein residues and RNA nucleotides were selected to define the distance monitored by SuMD during the two supervision phases.

A first attempt was made where Supervision Step-1 was initially applied to residues located within the folded regions of the proteins; in particular, the center of mass of Lys756 was considered as representative of the folded region, and U149 (interacting with Lys756 in the bound state) was chosen on the RNA counterpart. The resulting trajectories led to final states where the conformation of the structured domain was very different from the experimental X-ray structure (Table S2), and, moreover, the unstructured domain was collapsed and not prone to progress into the recognition of the major groove of RNA. On the other hand, these trajectories consistently indicated that the first protein–RNA contacts were predominantly mediated by the intrinsically disordered, positively charged segments. As shown in Figure S2A (Supporting Information), for the SF3A1-U1-SL4 system, the earliest contacts occurred through the disordered tail in Run2 and Run3, while in Run1, the disordered and folded regions approached the RNA nearly simultaneously. These observations suggested that, despite initial supervision being applied to structured regions, the intrinsically disordered segments play a dominant role in initiating recognition. Consequently, the supervision protocol was redesigned to monitor the approach of the disordered region to RNA during Supervision Step-1. This revised strategy resulted in a clearer and more consistent early interaction pattern, as illustrated in Figure S3A, where supervision applied directly to the disordered regions led to a well-defined initial contact phase across replicates for both systems. Together, these preliminary analyses supported the adoption of a two-step supervision scheme in which the disordered region drives the initial recognition (Supervision Step-1), followed by the supervision of the structured domains (Supervision Step-2) to capture subsequent stabilization events, as detailed below.

For Supervision Step-1, the focus was thus placed on the intrinsically disordered RGGR motif (Arg788, Gly789, Gly790, and Arg791), which was guided toward the center of mass defined by nucleotides G141, U145, and G154. These nucleotides were identified as interaction hotspots near the RGGR tail, both in the classical MD simulation described above and in the reference structure. In Supervision Step-2, the selection was shifted to Lys756 and nucleotide U149, another key contact observed in the classical MD trajectory and highlighted in Figure 1C. This second supervision phase was designed to promote the correct orientation of the folded globular domain after the initial engagement of the disordered tail.

Starting from the same initial configuration, three independent SuMD simulations were performed under identical Supervision Step-1 and Step-2 conditions. In particular, three independent runs of Supervision Step-1 were conducted, leading to the approach of the unstructured domain to its experimental bound conformation (Table S2). A second SuMD run was started from the last frame of these three independent replicates under Supervision Step-2 conditions. Notably, despite starting from the same initial configuration and applying identical supervision criteria, the three replicates extensively sampled different relative orientations of the RNA with respect to the protein (Figure 2B–D), exploring both the major and minor grooves.

**Figure 2:**
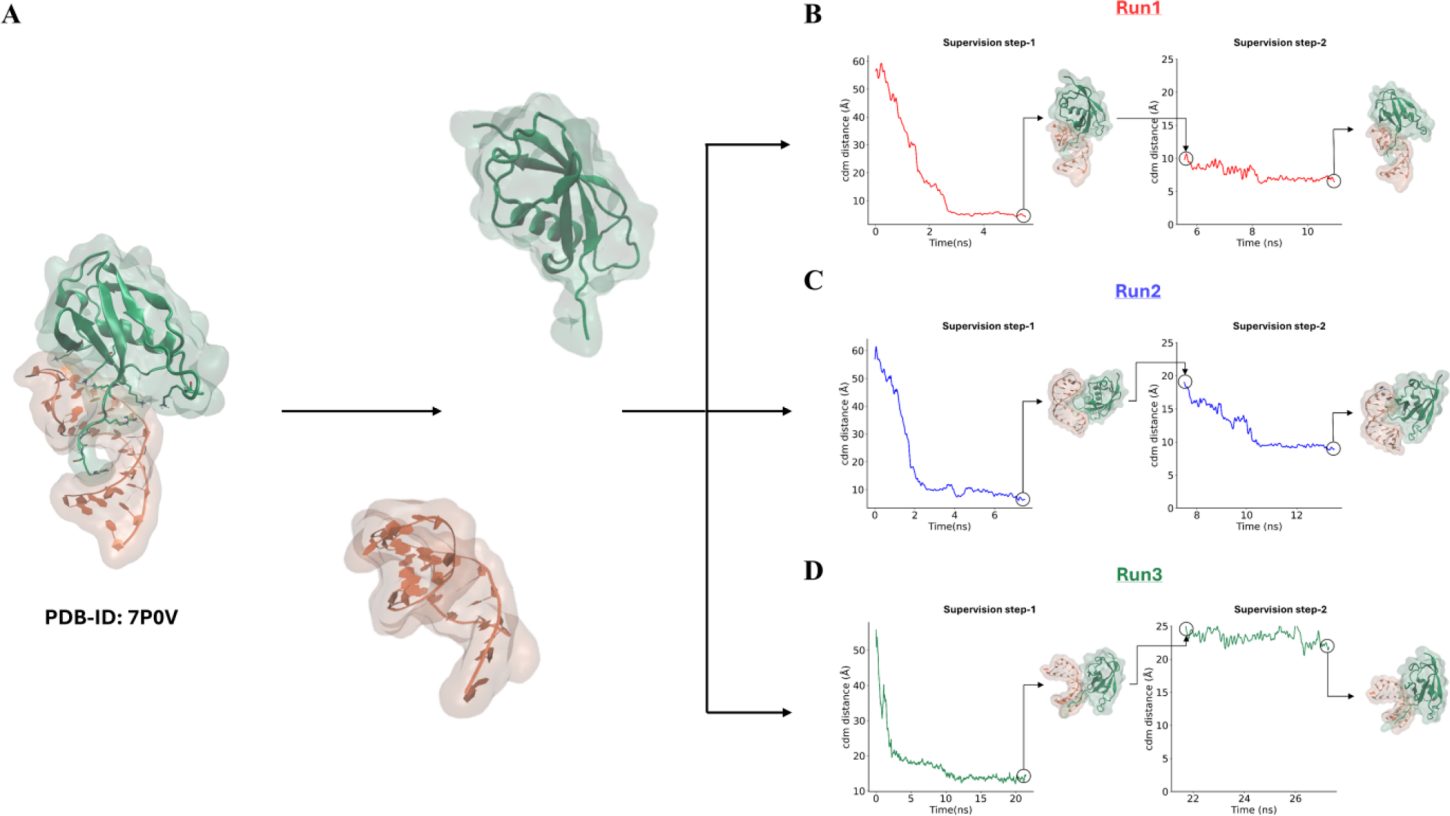
(A) Structural representation of the reference complex and schematic separation of the protein (green) and RNA (orange) used as the starting configuration for SuMD simulations. (B, D) Time evolution of the supervised center of mass distance for Run1 (B), Run2 (C), and Run3 (D) during Supervision Step-1 and Step-2. For each replica, the distance profiles illustrate the progressive reduction of the monitored center of mass distance during Step-1, followed by the second supervision phase guiding the final approach. The circled points indicate the last frame of each supervision step, while the corresponding structural snapshots highlight the relative orientation of protein and RNA at the end of each phase.

The final states obtained from the 2-Step SuMD simulations were compared to the experimental X-ray structure by aligning the nucleic acid backbone and computing the RMSD of the protein backbone. These results are collected in Table 1, with Run1 emerging as the trajectory achieving the closest positioning relative to the reference PDB structure.

**Table 1:** Summary of RMSD Values between Last Frames of the SuMD Simulations (Step-2) and X-ray Structures (Protein Backbone, after Alignment on the RNA Backbone)

	RMSD last_S2 VS X-ray	
	SF3A1	FUS
Run1	4.2 Å	38.25 Å
Run2	33.64 Å	14.31 Å
Run3	27.61 Å	12.41 Å

The reproduction of the X-ray complex with an RMSD of 4.2 Å proved the capability of the two-step SuMD approach to generate a near-native pose for the SF3A1-RNA complex. Subsequently, the possibility of discriminating Run1 from decoys (Run2 and Run3) was assessed to question the prospective applicability of this approach to predict RNA-IDR complex structures. In this context, the stability of the obtained complexes was compared by subjecting the last frames of the different replicates to classical MD simulations. The aim was to verify whether the near-native pose obtained at the end of the full recognition process (SuMD Step-2) was geometrically and energetically more stable during MD simulations as compared to decoys. In addition, the stability of recognition process intermediate states, obtained after SuMD Step-1, was also assessed, aiming to verify if it is possible to prioritize recognition pathways leading to near-native bound states, considering the stabilization of the unstructured region upon binding, thus suggesting which SuMD Steps-1 to extend into SuMD Steps-2 in a prospective scenario.

The last frames of each supervision phase were extracted and subjected to an additional 100 ns of classical MD, where both geometrical stability and interaction energy were evaluated. Specifically, since during Supervision Step-1 the approach of the protein unfolded region to RNA is monitored, and thus a final productive state would ideally present the IDR reproducing its native-like conformation, the positioning of the disordered tail was assessed in terms of RMSD from the initial state and interaction energy. After Supervision Step-2, leading to the complete bound state of the complex, geometrical and energetic stabilities were instead evaluated for the entire protein. The results of these analyses for the three runs are reported in Table S3 and Figures S4 and S5 (Supporting Information). In terms of structural stability and interaction strength, Run1 showed the most favorable performance at the end of both Supervision Step-1 and Step-2. After Step-1, Run1 displayed the lowest average RMSD (3.35 ± 0.61 Å) and the most favorable protein–RNA interaction energy (−452.9 ± 31.4 kcal/mol), compared to Run2 (RMSD: 6.61 ± 1.87 Å; Energy: −336.9 ± 80.0 kcal/mol) and Run3 (RMSD: 15.78 ± 4.69 Å; Energy: −358.5 ± 71.8 kcal/mol). A similar trend was observed after Step-2. Run1 maintained a low RMSD (1.93 ± 0.89 Å) while achieving the most favorable interaction energy (−904.5 ± 52.2 kcal/mol). In contrast, Run2 showed a comparable RMSD (3.98 ± 1.22 Å) but a markedly less favorable interaction energy (−651.3 ± 71.3 kcal/mol), whereas Run3 exhibited substantial structural instability (RMSD: 23.57 ± 10.42 Å) together with a higher variability in interaction energy (−677.1 ± 138.0 kcal/mol). Thus, in this case, RMSD and energetic analysis of prolonged MD simulations starting from the last SuMD frames seem able to discriminate X-ray-like conformations of the complex from decoys.

The complete trajectory of the two supervision phases for Run1 is shown in Video S1 (Supporting Information). In this simulation, the full recognition process between the protein and RNA occurred in approximately 10 ns of simulation time. A schematic summary of the binding process is presented in Figure 3. The residues involved in the first supervision phase are visible from the earliest stages of interaction and remain stable throughout the simulation (Figure 3C,D). In contrast, residues participating in the second phase are engaged later, consistent with a sequential binding mechanism. In the final SuMD frame, the protein backbone exhibited an RMSD of 4.2 Å, relative to the reference structure. In contrast, the RGGR disordered tail, which is essential for binding, showed a substantially lower RMSD of 1.3 Å.

**Figure 3:**
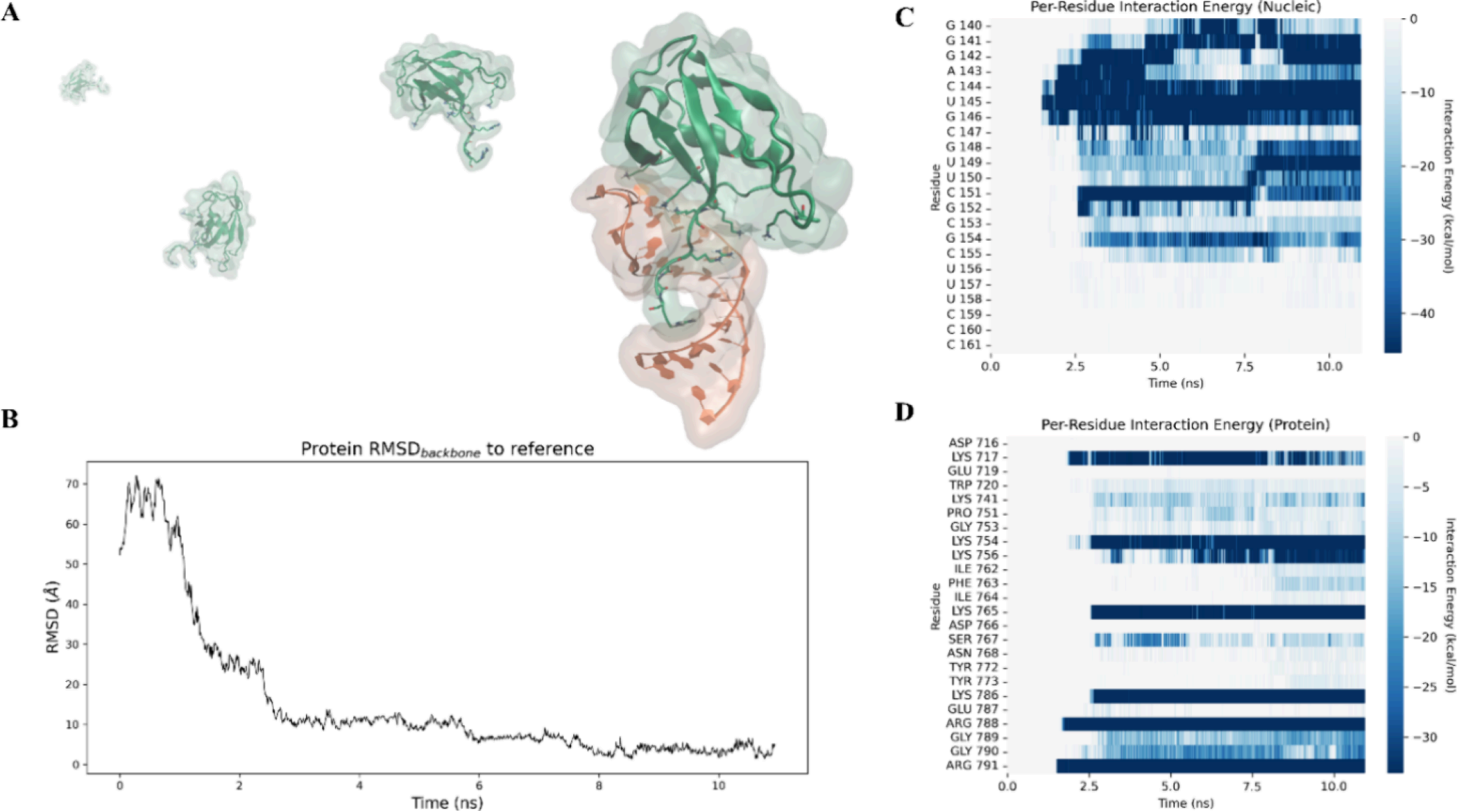
(A) Schematic representation of the SF3A1-U1-SL4 RNA–protein complex during the binding process. The protein is shown in green and trajectory, and the nucleic acid is in orange. (B) Backbone RMSD of the protein with respect to the crystallographic reference structure during the supervised molecular dynamics showing conformational convergence toward the native-like bound state. (C–D) Per-residue interaction energy heatmaps for the nucleic acid (C) and the protein (D) during the binding process. Interaction energies are color-coded from white (neutral) to dark blue (strongly favorable), with more negative values indicating stronger residue-level contributions to binding.

To assess the stability of the SuMD-generated complex from the selected Run1, the previously analyzed 100 ns classical MD trajectory was extended to 500 ns, and the same analyses performed on the experimental reference structure were applied. As shown in Figure 4B, the system maintained a stable geometry, with both the protein and RNA backbone RMSD values ranging between 1 and 3 Å, closely matching the reference crystal dynamics (Figure 4E). Similar trends were observed in the interaction profiles over time (Figure 3C,F). The energetic contributions of RNA and protein were comparable to those obtained from the experimental complex simulation. Collectively, these results confirm the ability of SuMD to reproduce the native binding mode, in terms of both structural alignment and interaction energetics, thereby providing a reliable basis for its application to the more complex RGG-containing system.

**Figure 4:**
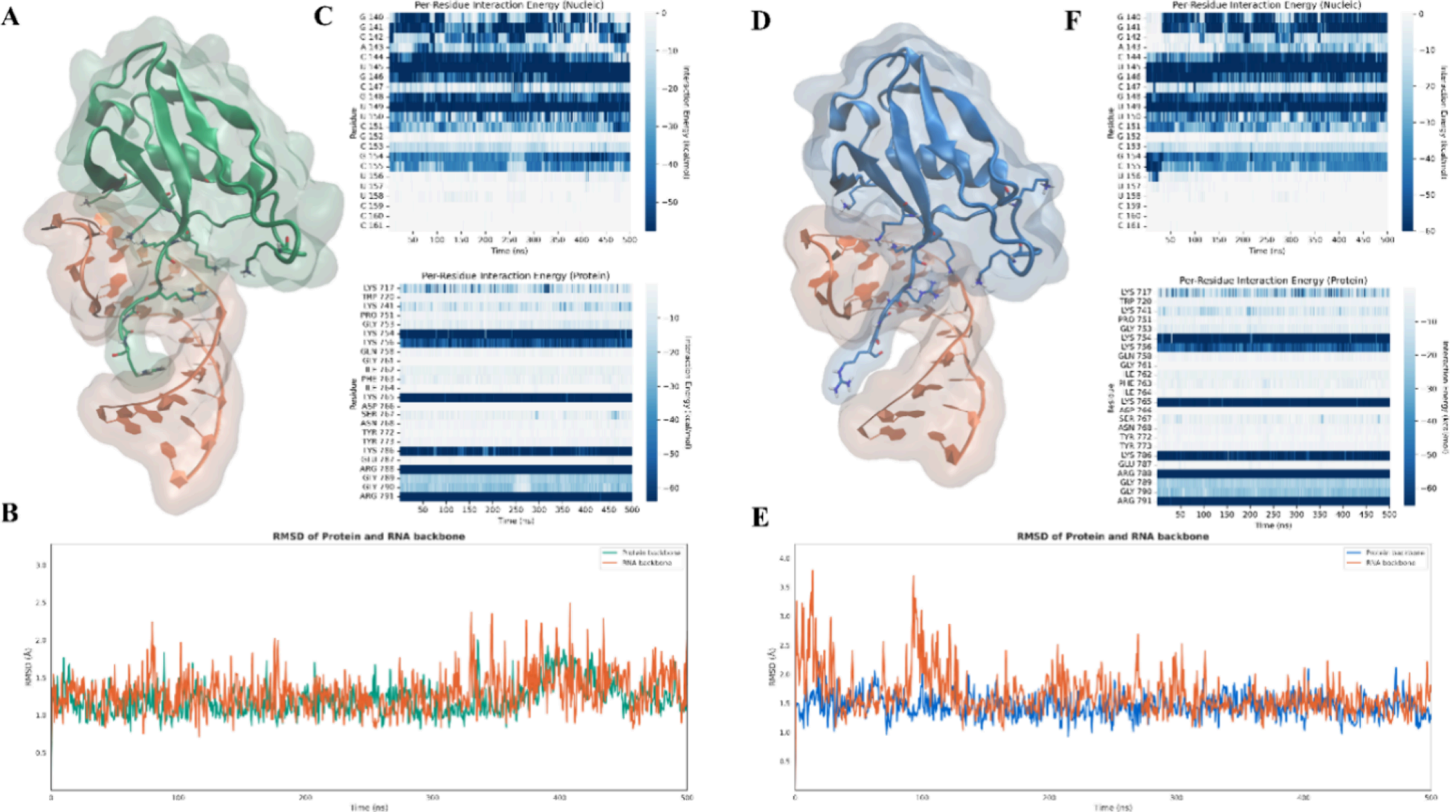
(A) Structural representation of the SF3A1-U1-SL4 complex obtained via SuMD: the protein is shown in green and the RNA is shown in orange. (B) RMSD of protein (green) and RNA (orange) backbones over 500 ns of classical MD simulation starting from the SuMD-generated complex. (C) Per-residue interaction energy profiles for the nucleic acid (top) and protein (bottom) during the SuMD-derived complex simulation. Darker shades of blue represent more favorable interaction energies. (D) Structural representation of the crystallographic reference complex: the protein is shown in blue and the RNA in orange. (E) RMSD of protein (blue) and RNA (orange) backbones over 500 ns of classical MD simulation of the reference structure. (F) Per-residue interaction energy profiles for the nucleic acid (top) and protein (bottom) during the simulation of the crystallographic complex.

### U1 snRNA Stem-Loop 3 in Complex with FUS/TLS RNA Recognition Motif (PDB ID: 6SNJ)

The human FUS (fused in sarcoma) protein is a multifunctional DNA/RNA-binding protein and a member of the FET family, which includes FUS, EWSR1, and TAF15.^85^ Also known as TLS (Translocated in Liposarcoma), FUS is involved in several cellular processes, including transcriptional regulation, pre-mRNA splicing, RNA transport, DNA damage response, and homologous recombination.^86^ It comprises two main functional domains: an N-terminal low-complexity region enriched in QGSY and glycine residues, which mediates liquid–liquid phase separation (LLPS) and protein–protein interactions,^87^ and a C-terminal nucleic acid-binding region containing an RNA recognition motif (RRM) and a zinc finger (ZnF), both flanked by RGG-rich sequences.^88^ Among these, the RRM domain has recently emerged as a key mediator of RNA binding. In 2019, the first NMR structure of the FUS RRM bound to a 23-nucleotide RNA stem-loop was published.^88^ This was followed in 2020 by a second NMR structure of the RRM bound to SL3 of U1 snRNA, a physiologically relevant RNA target within the spliceosome (Figure 1A).^16^ Importantly, this structure also features three RGG repeats inserted into the RNA minor groove, offering direct insight into their role in modulating RNA recognition through disordered, positively charged interactions. This structure provided critical insight into how disordered RGG repeats shape RNA recognition. The NMR model reveals that the 3′ YNY motif (U105, G106, and U107) establishes stable contacts with the RRM β-sheet surface. Additionally, the extended α1-β2 loop, which contains charged residues such as Lys312, Lys315, and Lys316, reinforces binding through major groove interactions. Significantly, the RGG repeats insert into the RNA minor groove, forming a flexible network of positively charged contacts that appear to modulate the specificity and stabilize the complex. This distinctive interaction mode extends the canonical view of RRM-RNA recognition, suggesting that disordered regions can act as adaptable modulators of RNA binding.

To assess the conformational stability and interaction dynamics of the NMR-derived FUS-SL3 reference structure, we performed a 500 ns classical MD simulation. Compared with the smaller and more rigid SF3A1 test case, this system is larger and structurally more complex, including a 14-residue intrinsically disordered region containing three RGG repeats. Despite its inherent flexibility, this complex remained stable throughout the trajectory, with backbone RMSD values for both protein and RNA fluctuating around 5 Å, with occasional peaks slightly above 6 Å (Figure 5A).

**Figure 5:**
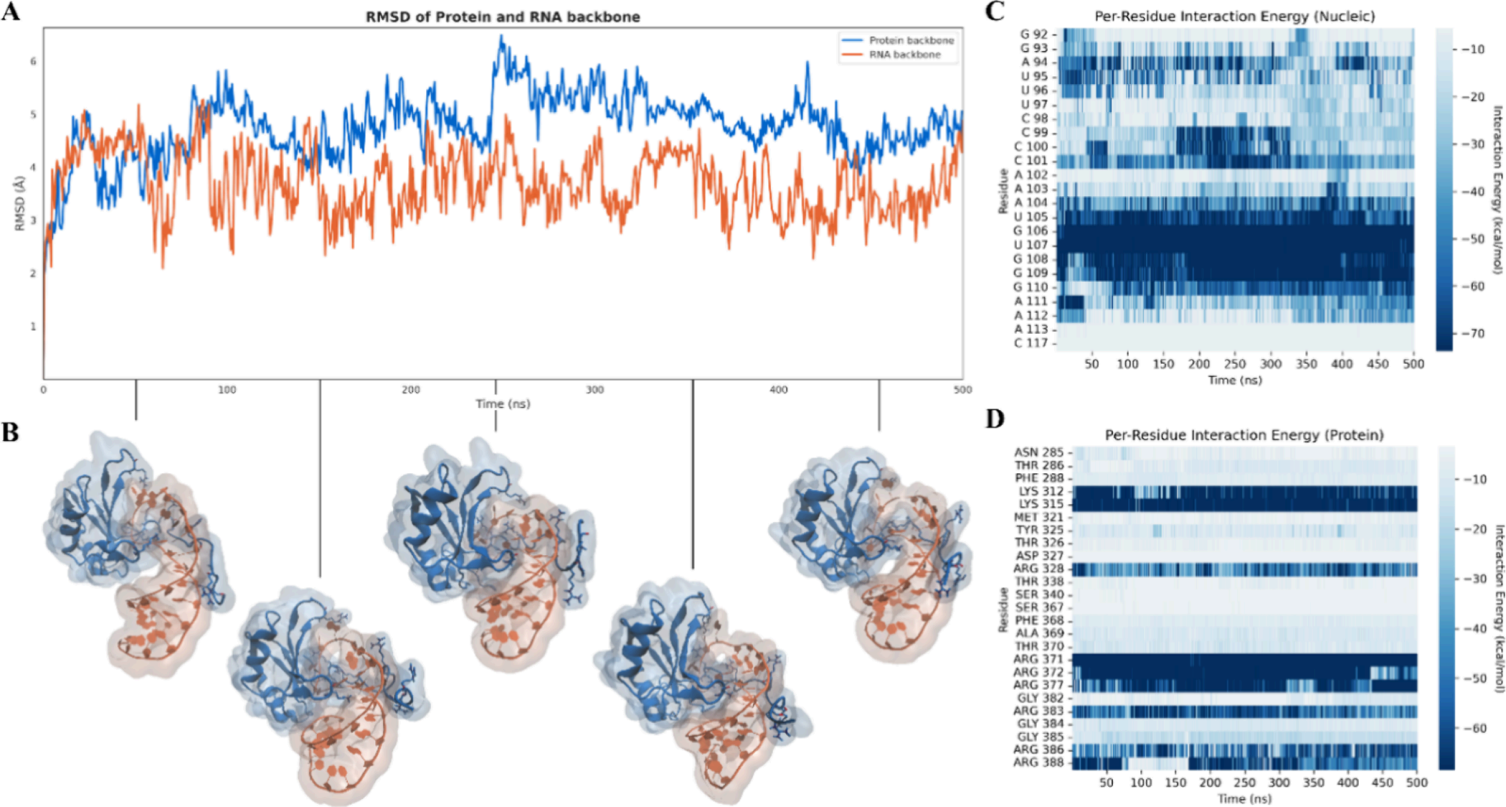
(A) Root-mean-square deviation (RMSD) of the protein FUS (blue) and RNA U1-SL3 (orange) backbones during the molecular dynamics simulation, indicating the overall structural stability of the complex. (B) Representative conformations extracted at selected time points, illustrating the relative orientation and conformational stability of the protein (blue) and RNA (orange) throughout the trajectory. (C, D) Per-residue interaction energy profiles for the nucleic acid (C) and protein (D) components computed over the course of the simulation, highlighting the key residues contributing to complex stability in the crystallographic model.

Consistent with these observations, the classical MD simulation of the reference complex further highlighted the energetic dominance of positively charged protein residues. All arginines within the disordered region exhibited markedly favorable interaction energies, while the structured portion of the protein contributed additional stabilizing contacts through several lysines. These residues also emerged as key determinants of the interaction throughout the simulation, in agreement with the findings reported in the reference study. A similar trend was observed on the RNA side: nucleotides A104 to G110 formed a persistent and energetically favorable cluster, representing the core segment of the RNA that engages the protein across both the minor and major grooves.

Based on this trajectory analysis, key RNA-interacting residues were selected to define the two-step SuMD supervision protocol. Analogously to the previous case study, after an unproductive attempt to first supervise the structured domain approach to RNA (Table S2 for RMSD values), the supervision strategy was designed to first promote engagement of the disordered region and subsequently stabilize the structured portion of the protein. In Supervision Step-1, the distance between the center of mass of nucleotides U105, G106, U107, G108, G109, and G110 on RNA and the center of mass of the six arginines (Arg371, Arg372, Arg377, Arg383, Arg386, and Arg388) of the disordered region was monitored, with a rapid engagement of the RNA minor groove, and the IDR acting as an initial anchoring interface. In Supervision Step-2, the approach of a lysine-rich loop (Lys312, Lys315, and Lys316) to the major groove of RNA (U105, G106, U107, G108, G109, and G110) was monitored.

Starting from the same initial configuration, three independent SuMD simulations were performed under identical Supervision Step-1 and Step-2 conditions, consistent with the protocol applied in the previous case. Despite the identical starting point and supervision criteria, the three replicates explored distinct interaction modes and relative RNA orientations as highlighted by RMSD values from the experimental NMR complex reported in Table 1, reflecting a broad conformational landscape rather than convergence toward a single identical geometry. This behavior indicates that the shared starting configuration did not impose a bias but instead allowed extensive sampling of energetically favorable recognition pathways. Among the three replicates, Run3 was the one conducting the NMR-most similar pose.

As in the previous case study, to verify the possibility of objectively distinguishing and highly ranking Run3 with respect to the others, the stability of the complexes obtained through SuMD was examined. The last frames of Supervision Step-1 and Step-2 were submitted to an additional 100 ns of classical MD to assess their stability. At the end of Step-1, RMSD from the initial state and interaction energy were evaluated, focusing on the disordered segment (residues 376–390), whereas after Step-2, the analyses were extended to the entire protein to verify stabilization of both the disordered and structured regions. The corresponding RMSD from the initial state and interaction energy profiles for all replicates are reported in Supporting Information (Table S3 and Figures S6 and S7).

Following Supervision Step-1, Run3 displayed the lowest average RMSD (4.80 ± 0.60 Å) of the unstructured region, indicating comparatively higher structural stability relative to Run1 (7.30 ± 0.80 Å) and Run2 (7.20 ± 0.60 Å), which both fluctuated around higher RMSD values. From an energetic standpoint, Run3 also exhibited the most favorable protein–RNA interaction energy (−590.00 ± 70.00 kcal/mol), followed by Run2 (−520.00 ± 60.00 kcal/mol), whereas Run1 showed overall less favorable interaction energies (−380.00 ± 40.00 kcal/mol). After Supervision Step-2, Run3 presented the lowest RMSD values (6.00 ± 1.60 Å), comparable to Run1 (6.09 ± 1.69 Å) and lower than Run2 (8.69 ± 3.04 Å), while displaying the most favorable interaction energy among the three replicas (−992.10 ± 110.30 kcal/mol), compared to Run1 (−835.20 ± 91.10 kcal/mol) and Run2 (−660.80 ± 98.60 kcal/mol). Taken together, the combined structural and energetic metrics across both supervision steps indicate that Run3 provides the most stable and energetically favorable configuration overall, supporting its identification as the productive trajectory for this case study.

In Video S2 (Supporting Information), the complete Run3 selected is available. Analysis of the per-residue interaction energy profiles of this simulation, evaluated on both the nucleic acid side (Figure 6C) and the protein side (Figure 6D), further supports the mechanistic picture described above. In particular, clear initial engagement of Arg377, Arg383, and Arg388, located within the disordered tail, can be observed during the early stages of recognition. Only at a later stage is the interaction visible for Lys312 and Lys316, positioned within the loop of the globular domain, consistent with the second supervision phase guiding the progressive stabilization of the complex.

**Figure 6:**
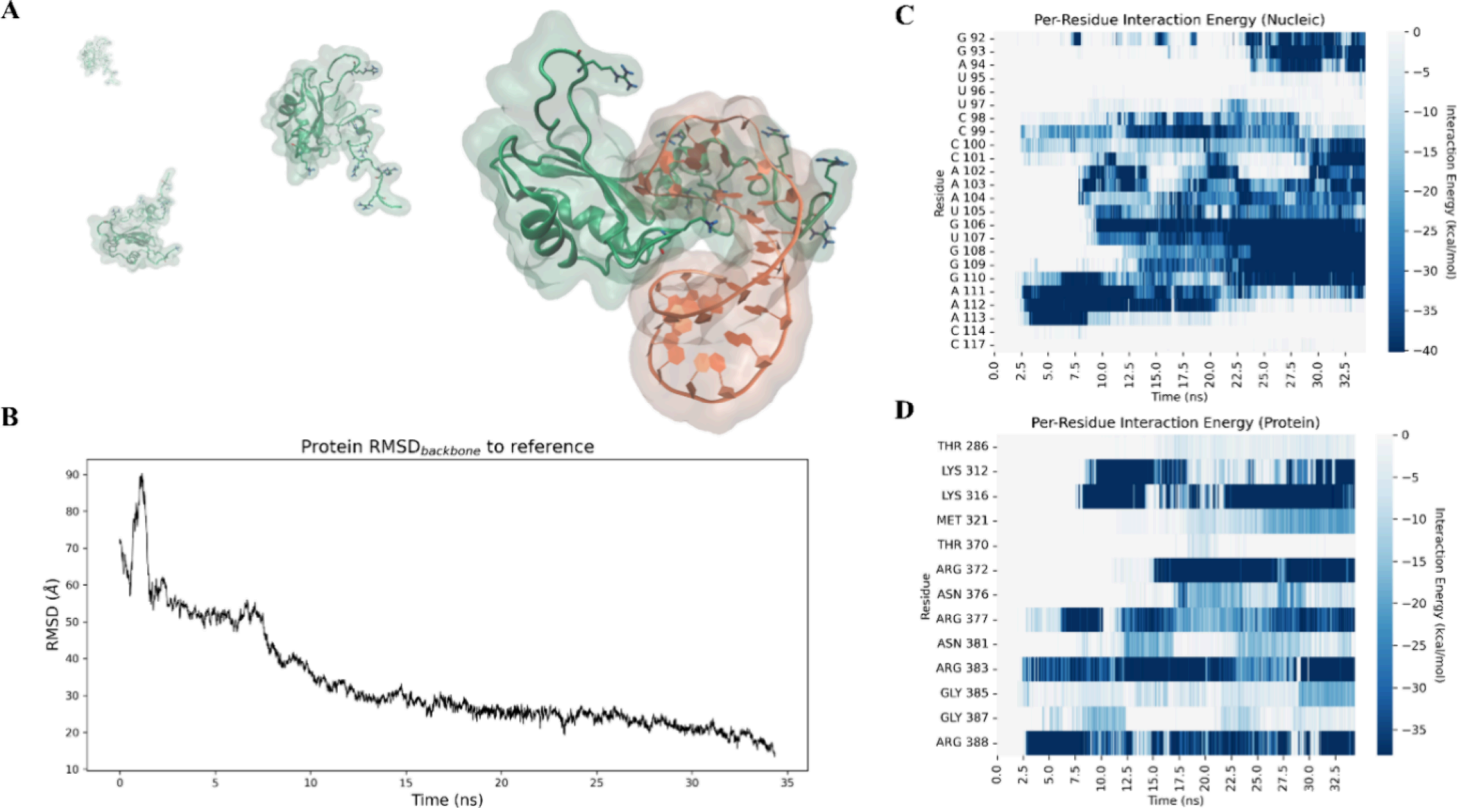
(A) Schematic representation of the FUS-U1-SL3 RNA–protein complex during the binding process. The protein is shown in green and trajectory, the nucleic acid in orange. (B) Backbone RMSD of the protein with respect to the crystallographic reference structure during the supervised molecular dynamics showing conformational convergence toward the native-like bound state. (C, D) Per-residue interaction energy heatmaps for the nucleic acid (C) and the protein (D) during the binding process. Interaction energies are color-coded from white (neutral) to dark blue (strongly favorable), with more negative values indicating stronger residue-level contributions to binding.

In the final SuMD frame, the protein backbone showed an RMSD of 12.4 Å relative to the reference, whereas the disordered RGG-containing tail displayed a lower RMSD of 6.3 Å. These higher RMSD values, as compared to the SF3A1-U1-SL4 test case, are indicative of the intrinsically greater flexibility of FUS. During the early recognition phase of the selected SuMD trajectory, prior to the establishment of stable contacts with the RNA, the extended disordered tail (residues 376–390) exhibited pronounced conformational plasticity. Compared with the shorter disordered segment analyzed in the previous study, this longer region explored a substantially wider conformational space. Monitoring the RMSD of the disordered tail relative to the initial SuMD frame during the frames preceding the first protein–RNA contact revealed a mean RMSD of 17.2 Å and maximum deviations of up to 20.4 Å. These fluctuations reflect the high intrinsic flexibility of the tail, while the protein remains in the bulk solvent, emphasizing the dynamic nature of the recognition process. Consistently, the Cα-RMSF was computed over the 500 ns classical MD simulations of the reference experimental structures of the two case studies (Figure S1), revealing a higher overall flexibility for FUS as compared to SF3A1, further supporting the intrinsically dynamic nature of this binding event. This observation agrees with the experimental NMR ensemble of the FUS-U1-SL3 complex, where the RGG-containing tail exhibits substantial conformational heterogeneity (average RMSD ≈ 5.5 Å, peaks up to ∼10.7 Å among deposited conformers).

Nevertheless, the near-native binding pose across key geometric interaction regions (Figure 7), together with the strong similarity in the RNA–protein interaction energy profiles, confirms the robustness of SuMD in modeling highly dynamic RNA–protein assemblies.

**Figure 7:**
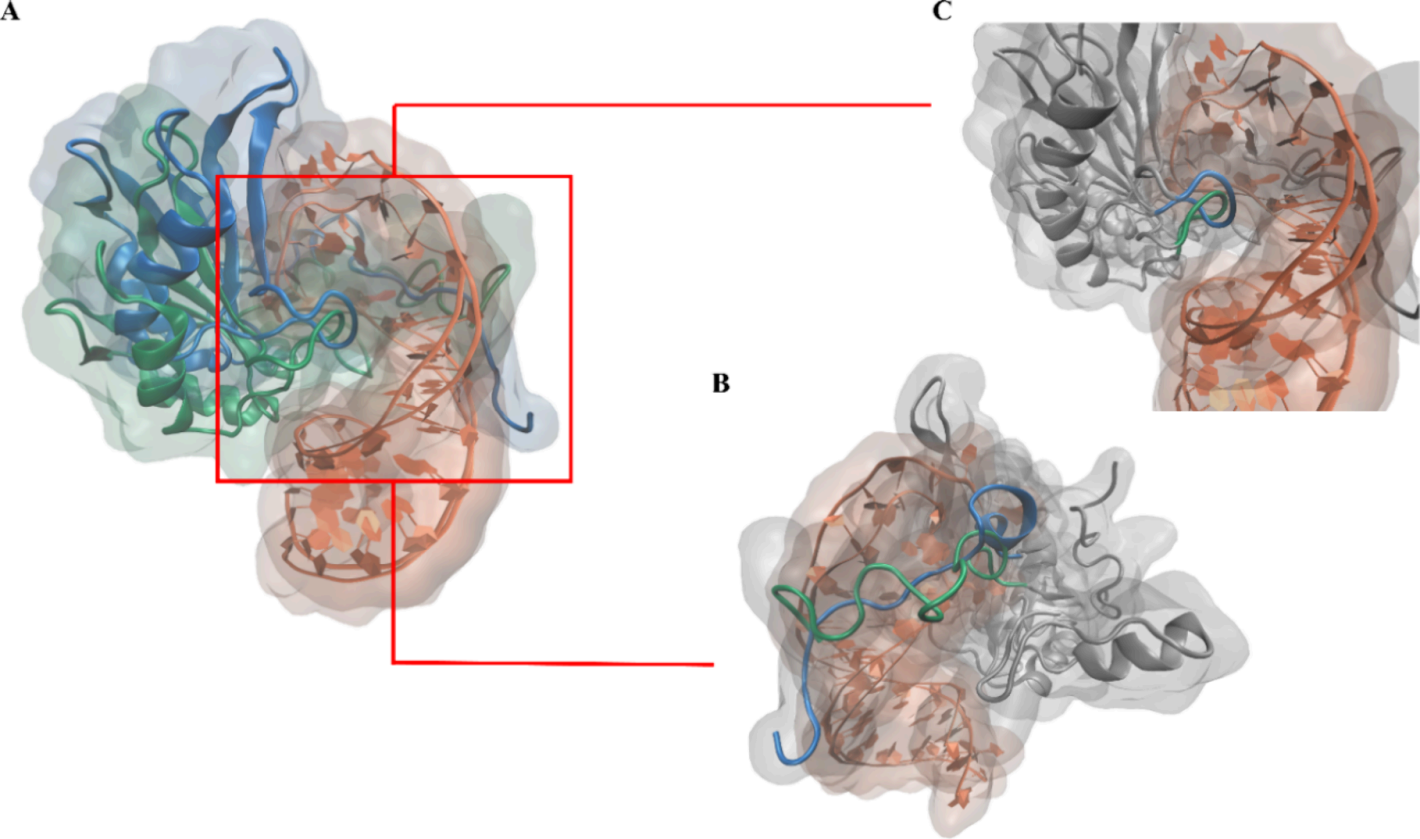
(A) Structural superposition of the crystallographic complex (PDB ID: 6SNJ), representative of the FUS-U1-SL3 interaction, with the last frame of the SuMD simulation. The FUS protein from the reference structure is shown in blue, while the FUS conformation obtained from the SuMD simulation is shown in green. The RNA molecule is depicted in orange. (B) Focus on the interaction of the disordered sequence containing the RGG repeats within the minor groove of the RNA. (C) Close-up view highlighting the interaction between the RNA loop and lysine residues located in the major groove.

As in the previous case, the SuMD-derived complex was further simulated by extending the previously analyzed 100 ns classical MD trajectory to a total length of 500 ns, and the resulting trajectory was compared with the one initiated from the experimental NMR structure. The resulting RMSD and per-residue interaction energy profiles closely resembled those obtained from the reference simulation (Figure 8). In both trajectories, nucleotides 105–110 consistently exhibited the strongest favorable interaction energies, engaging with the β-sheet, the α1-β2 loop, and the disordered RGG-containing region, where they interact with key arginine and lysine residues discussed previously (Figure 8C,F).

**Figure 8:**
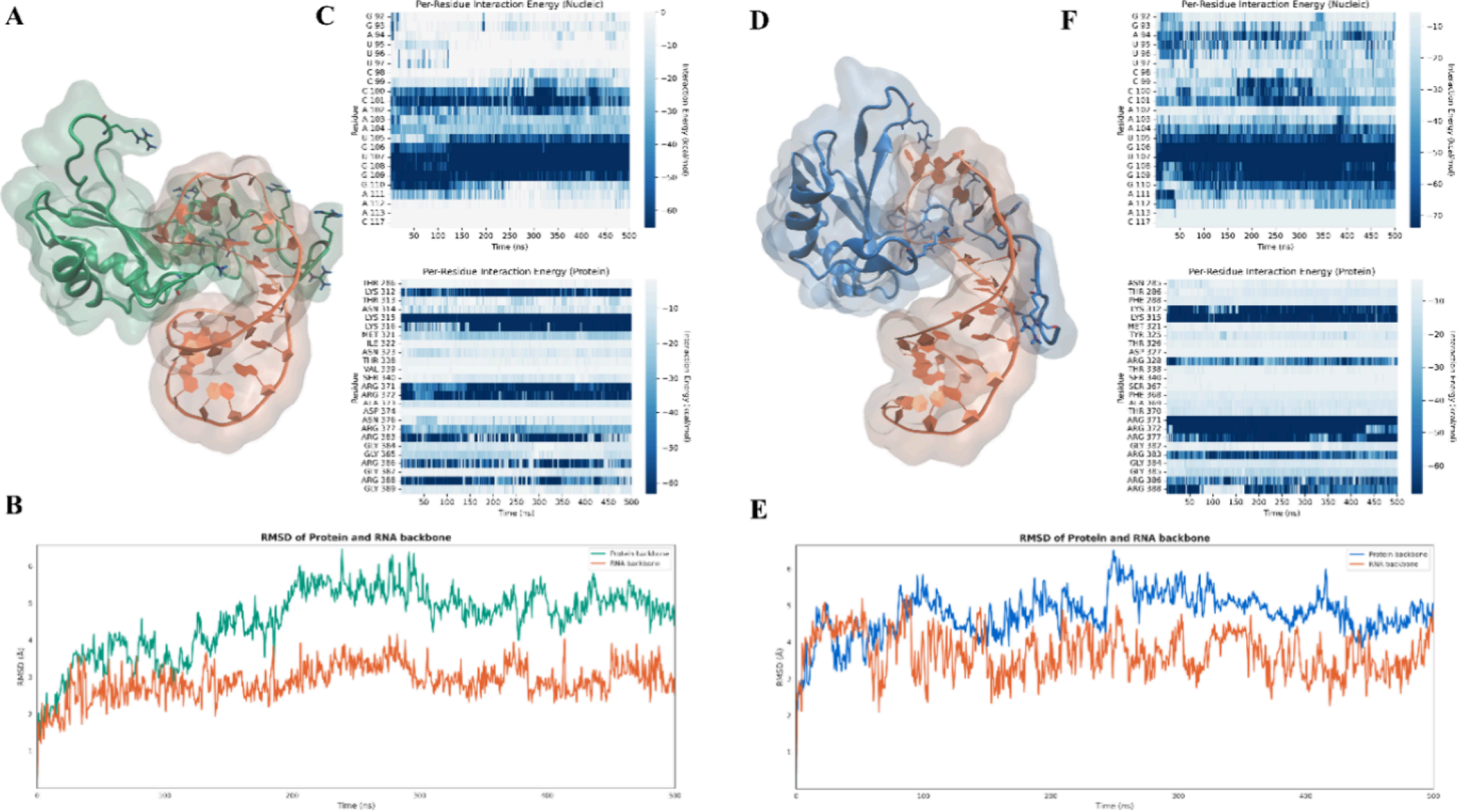
(A) Structural representation of the FUS-U1-SL3 complex obtained via SuMD: the protein is shown in green and the RNA in orange. (B) RMSD of protein (green) and RNA (orange) backbones over 500 ns of classical MD simulation starting from the SuMD-generated complex. (C) Per-residue interaction energy profiles for the nucleic acid (top) and protein (bottom) during the SuMD-derived complex simulation. Darker shades of blue represent more favorable interaction energies. (D) Structural representation of the crystallographic reference complex: the protein is shown in blue and the RNA in orange. (E) RMSD of protein (blue) and RNA (orange) backbones over 500 ns of classical MD simulation of the reference structure. (F) Per-residue interaction energy profiles for the nucleic acid (top) and protein (bottom) during the simulation of the crystallographic complex.

### Telomeric Repeat-Containing RNA 12 (TERRA12, PDB ID: 2KBP) in Complex with Small EDRK-Rich Factor 2 (SERF2, PDB ID: 9DT0)

SERF-related proteins (Small EDRK-rich Factor) are small (60–80 amino acids), highly charged polypeptides characterized by a conserved N-terminal domain and originally identified as in vivo modulators of amyloid formation associated with age-related diseases.^89,90^ Within this family, human SERF2 is an intrinsically disordered protein that has recently emerged as a specific binder of RNA G-quadruplexes (rG4s), noncanonical tetrahelical RNA structures formed by guanine-rich sequences through Hoogsteen base pairing.^91^ rG4s are increasingly recognized as functional regulatory elements in processes such as telomere maintenance, stress response, and translational control.^92^ Recent biophysical and structural studies demonstrated that SERF2 binds telomeric rG4s, including TERRA-derived sequences, with low micromolar affinity and forms dynamic complexes capable of promoting phase separation under crowding conditions, highlighting its potential role in ribonucleoprotein condensate formation.^91^

In that recent study, the interaction between SERF2 and telomeric rG4s of different lengths (TERRA10, TERRA12, and TERRA23) was extensively characterized, revealing selective binding, multimeric complex formation, and liquid–liquid phase separation behavior. Although the employed methodologies did not allow the determination of an experimental 3D structure of the complex, key nucleotides involved in the interaction were identified through NMR-HSQC techniques, particularly G4, G5, G9, and G10 in the TERRA10 and TERRA12 systems, as well as the most relevant residues within SERF2, enabling a distinction between primary and secondary binding determinants.

The absence of an experimentally resolved complex structure, together with the availability of the individual components, SERF2 (PDB ID: 9DT0) and TERRA12 (PDB ID: 2KBP), made this system particularly suitable for an exploratory prospective application of the two-step SuMD methodology, following the strategy adopted in the two retrospective case studies described above. Although SERF2 is not an RGG/RG-containing protein, it is highly disordered, featuring positively charged N-terminal (residues 1–32) and C-terminal (residues 48–56) regions separated by a short helical core (residues 37–47). Experimental evidence indicates that the N-terminal region represents the primary RNA-binding interface, while the C-terminal segment contributes to subsequent stabilization of the complex.^91^

These structural and functional insights guided the two-step SuMD protocol. Initial simulation conditions were set as described in the previous case studies. During Supervision Step-1, the supervision was driven by monitoring the distance between the center of mass of the N-terminal region and the center of mass of RNA nucleotides G4, G5, G9, and G10. Upon completion of this first phase, Supervision Step-2 was initiated, monitoring the approach of the C-terminal region toward the RNA. In this case, supervision was based on the center of mass of the entire nucleic acid, consistent with the experimentally observed less specific interaction of that region.

Under these supervision conditions, SuMD simulations were performed in triplicate. For each replicate, the last frames of Supervision Step-1 and Step-2 were extended by 100 ns of classical MD, and average protein backbone RMSD values together with protein–RNA interaction energy profiles were evaluated using the same protocol described previously. The results for all three runs are reported in the Supporting Information (Figures S8 and S9, Table S3). Following Supervision Step-1, Run3 displayed the lowest average RMSD (5.07 ± 1.11 Å) of the N-terminal region together with the most favorable interaction energy (−538.20 ± 77.40 kcal/mol) compared to Run1 (RMSD: 5.86 ± 1.11 Å; energy: −494.80 ± 86.00 kcal/mol) and Run2 (RMSD: 6.55 ± 0.93 Å; energy: −489.80 ± 88.70 kcal/mol), indicating slightly improved stability at this stage of the recognition process. However, after Supervision Step-2, Run1 exhibited the lowest RMSD (5.20 ± 1.18 Å) and the most favorable interaction energy (−569.60 ± 74.30 kcal/mol), whereas Run2 showed higher structural fluctuations (8.09 ± 2.49 Å) and less favorable interaction energies (−528.50 ± 76.50 kcal/mol). Run3 displayed the highest RMSD (11.96 ± 3.53 Å) together with the least favorable interaction energy (−430.20 ± 64.00 kcal/mol), indicating reduced stability following the second supervision phase. Taken together, the combined structural and energetic metrics across both supervision steps indicate that Run1 provides the most consistent and stable binding configuration overall, supporting its identification as the putative trajectory best describing the binding process among the sampled ones for this case study.

The trajectory of this simulation is provided in Video S3. The overall length of the SuMD simulation was approximately 18 ns, in line with the time scales observed in the previous case studies. A schematic recap of the complete binding process is shown in Figure 9.

**Figure 9:**
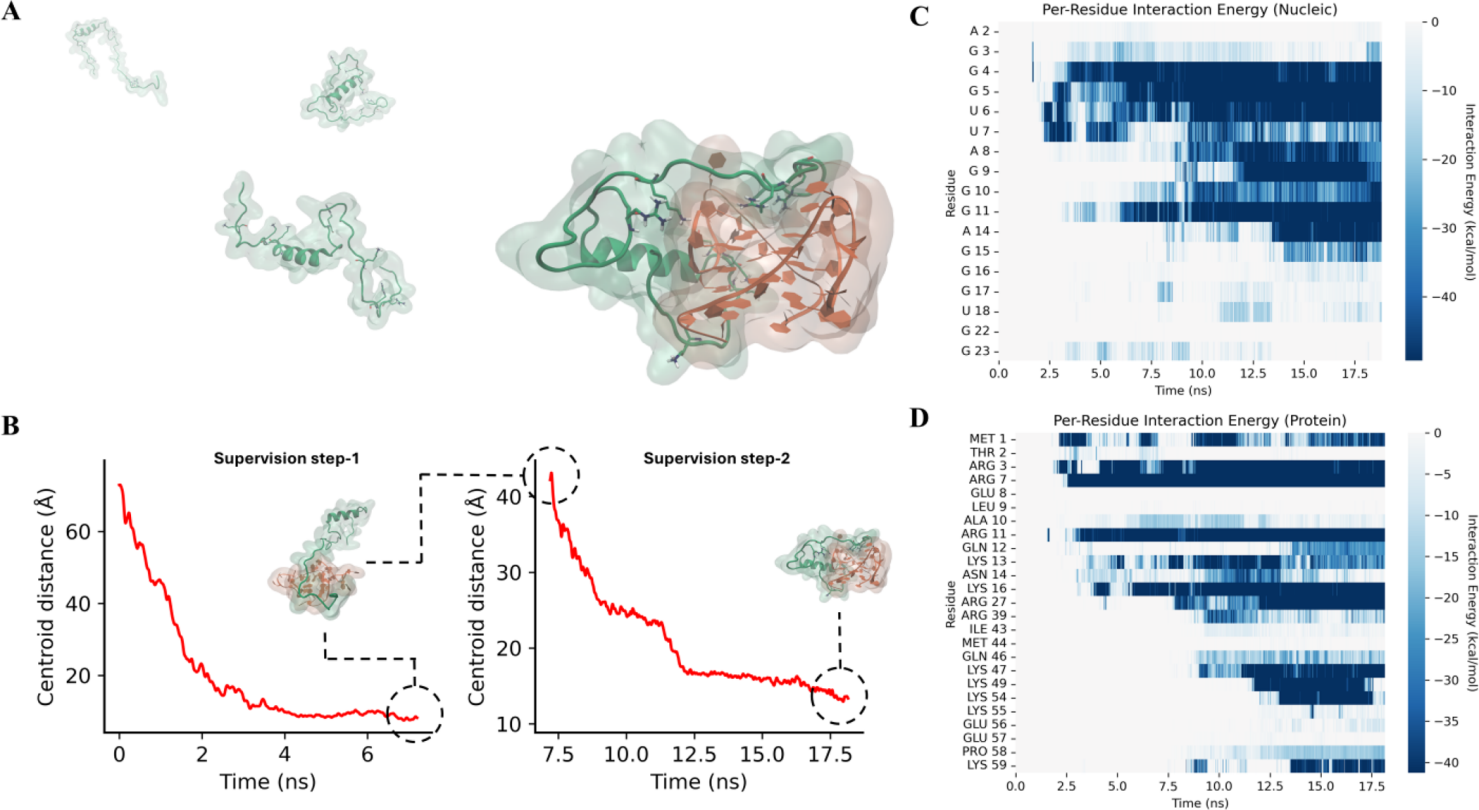
(A) Schematic representation of the SERF2-TERRA12 RNA–protein complex during the binding process. The protein is shown in green and trajectory, and the nucleic acid in orange. (B) Recap of the complete SuMD binding process, showing the supervised center-of-mass distance approaches for both Supervision Step-1 and Step-2. (C, D) Per-residue interaction energy heatmaps for the nucleic acid (C) and the protein (D) during the binding process. Interaction energies are color-coded from white (neutral) to dark blue (strongly favorable), with more negative values indicating stronger residue-level contributions to binding.

As observed by the previous systems, the per-residue interaction energy profiles (Figure 9C, nucleic acid; Figure 9D, protein) clearly reflect the two-step supervision strategy. An initial energetic contribution arises from the positively charged residues located in the N-terminal region (residues 1–16, highlighted in Figure 9D), which were also identified in the reference study as the most significantly perturbed in chemical shift analyses. Only in a later stage does a favorable energetic contribution emerge from residues in the C-terminal region (Gln46, Lys47, Lys49, and Lys54), consistent with the second supervision phase guiding the progressive stabilization of the complex.

Particularly remarkable is the per-residue interaction energy profile of RNA (Figure 9C). The nucleotides most strongly involved in the interaction are highly clustered within the G4–G11 region, which includes the key residues (G4, G5, G9, and G10) previously identified by NMR-HSQC chemical shift perturbation as critical determinants of rG4 recognition.^91^ Thus, the pose obtained as the last frame of SuMD Run1, selected as most stable among the sampled states, closely reflects the interaction pattern inferred from experimental data, resulting in a putative structural model of the SERF2-TERRA12 RNA–protein complex.

Following the same protocol applied in the previous case studies, the last frame of SuMD Run2 was subjected to the 500 ns classical MD simulation, extending the previously described 100 ns simulation, to assess the long-term geometric stability of both protein and RNA, as well as the persistence of key protein–RNA interactions through per-residue interaction energy analysis.

Visual inspection of the SuMD-derived structure (Figure 10A) shows that the positively charged N-terminal residues (displayed in stick representation) adopt a favorable orientation toward nucleotides G4, G5, G9, and G10 (displayed in stick representation), consistent with the predicted primary binding interface, as confirmed by per-residue electrostatic interaction analysis (Figure 10B). Geometric analyses (Figure 10D) indicate that the RNA remains structurally stable throughout the simulation, as reflected by steady RMSD values. Despite being almost entirely intrinsically disordered and highly enriched in positively charged residues, the protein also displays overall acceptable stability: its RMSD remains below ∼4 Å for approximately 350 ns of the 500 ns simulation, shows a transient increase up to ∼5 Å, and then returns below 4 Å until ∼450 ns. Only during the final 50 ns does the protein RMSD rise to ∼7 Å; however, considering its fully disordered nature and the absence of globular domains, this behavior can still be regarded as compatible with a stable bound ensemble.

**Figure 10:**
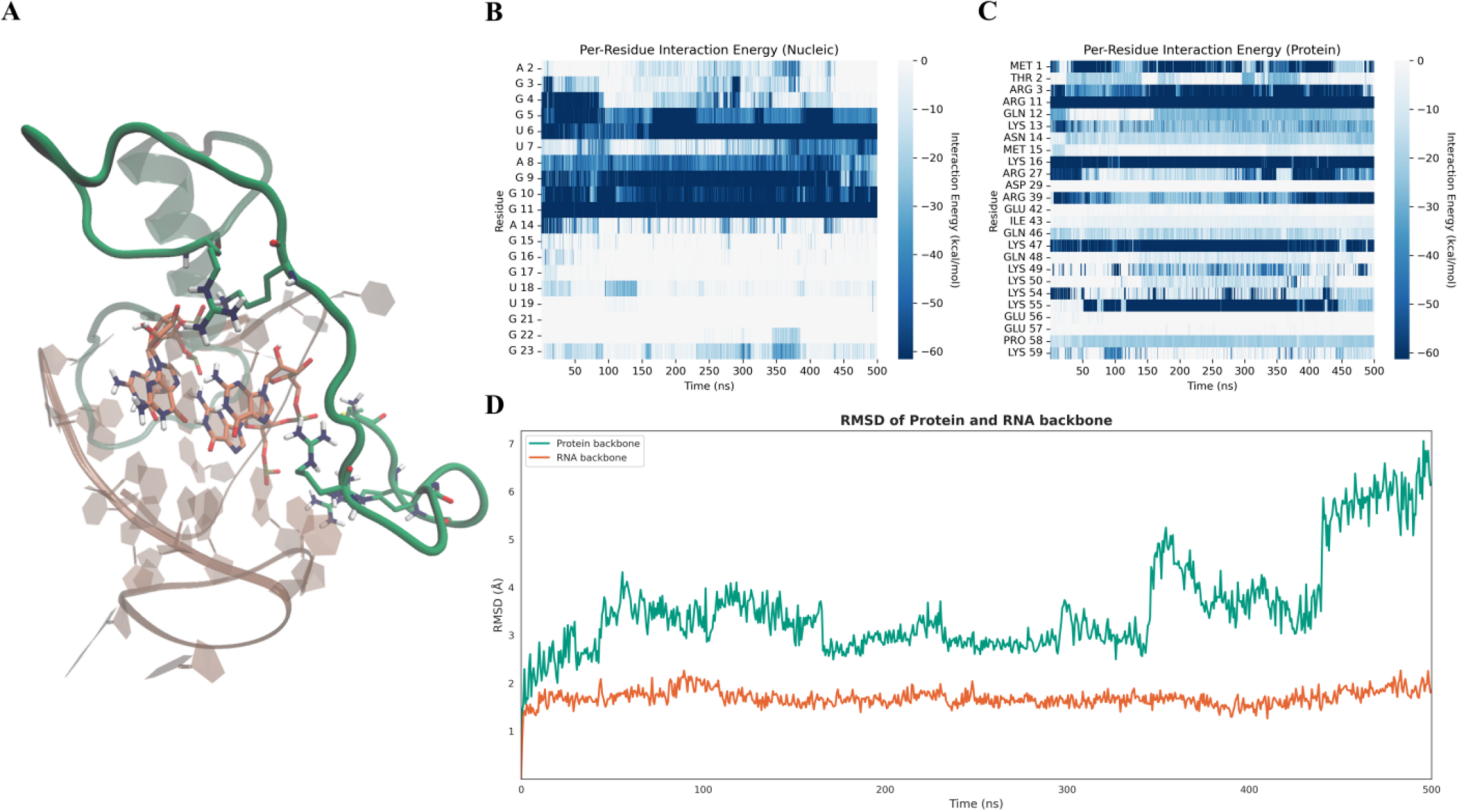
(A) Structural representation of the SERF2-TERRA12 complex obtained via SuMD. The protein is shown in green, and the RNA in orange. Key residues in the N-terminal of SERF2 are highlighted in stick representation, while nucleotides G4, G5, G9, and G10 of the RNA are also shown in stick to emphasize the primary contact region. (B) RMSD of protein (green) and RNA (orange) backbones over 500 ns of classical MD simulation, starting from the SuMD-generated complex. (C) Per-residue interaction energy profiles for the nucleic acid (top) and protein (bottom) during the SuMD-derived complex simulation. Darker shades of blue represent more favorable interaction energies.

Importantly, the per-residue interaction energy profiles for both the RNA (Figure 10B) and the protein (Figure 10C) remain consistent throughout the simulation, confirming that the selected SuMD-derived last frame represents a complex that is stable not only from a geometrical standpoint but also in terms of its key intermolecular interactions.

## Discussion and Conclusions

In this study, we report the first application of supervised molecular dynamics (SuMD) to investigate recognition processes between nucleic acids and intrinsically disordered proteins (IDPs), with a specific focus on proteins containing RGG domains or positively charged intrinsically disordered regions (IDRs). While previous applications of this technique focused primarily on well-folded protein–ligand or protein–RNA complexes, here, we extend its use to two retrospective RNA–protein systems characterized by pronounced conformational plasticity: the SF3A1-UBL domain bound to U1 snRNA stem-loop 4 (SL4), and the FUS RRM domain bound to U1 snRNA stem-loop 3 (SL3). These systems were selected not only for their structural and functional relevance in splicing regulation but also for the inherent challenges they present, as positive charges and flexible regions dominate their binding interfaces, often mediating recognition through transient, multistep mechanisms.

A two-step SuMD approach proved capable of capturing these complex recognition processes, reconstructing binding poses consistent with the experimentally determined structures, despite the presence of extended disordered elements. Each system was explored in triplicate to evaluate the variability of the recognition process: as reported in Table S3, independent runs produced diverse binding poses, reflecting the intrinsic conformational plasticity of RNA-IDR interfaces and the nondeterministic nature of techniques such as SuMD. While further increasing the number of replicas would, in principle, improve sampling coverage, the three runs adopted represent a pragmatic balance between accuracy and computational cost, as for both retrospective systems, at least one replica converged toward a pose in close structural agreement with the experimental reference, supporting the viability of this protocol.

The productive trajectory was selected based on objective criteria combining average RMSD values of the protein backbone and protein–RNA interaction energy profiles calculated from 100 ns classical MD extensions of the last frames of SuMD trajectories. This stability-based selection strategy allowed identification of the trajectory that most consistently preserved favorable geometric and energetic features rather than relying solely on structural proximity to the reference complex.

In the case of SF3A1-U1-SL4, this technique rapidly reproduced the native arrangement of the RGGR-containing tail and the associated network of lysine contacts after approximately 10 ns. On the other hand, the more structurally complex FUS-U1-SL3 assembly required a slightly longer time scale of around 35 ns to achieve a stable binding pose.

Building on this retrospective validation, the same SuMD protocol was subsequently applied to a prospective RNA-IDR system lacking an experimentally resolved complex structure, namely, the interaction between SERF2 and telomeric rG4 RNA, providing a putative model of the complex, consistent with experimental binding data.

The protocol, which included an initial SuMD posing phase followed by classical MD refinement, enabled the identification of residues involved in the binding process and reproduced the experimentally resolved complexes with close structural correspondence for SF3A1-U1-SL4 and a higher deviation for FUS-U1-SL3, consistent with the greater dynamic behavior and structural complexity of this system. Importantly, in the prospective SERF2-rG4 case, where no experimental complex structure is available, the same workflow yielded a binding mode consistent with residue-level experimental observations, including the enrichment of interactions on nucleotides identified by chemical shift perturbation analyses. The combined geometric and energetic analysis is particularly relevant for highly dynamic systems, where conformational flexibility and transient binding modes are evident both in simulations and in experimental structures. In such contexts, static reproduction of atomic coordinates alone is insufficient to fully describe the recognition mechanism. For this reason, residue-level interaction fingerprints were generated to monitor the temporal evolution of key energetic contributions and to compare them either with experimentally resolved complexes (retrospective systems) or with independent experimental information such as NMR-derived binding determinants (prospective systems).

The stability of SuMD-generated complexes was assessed in 500 ns classical molecular dynamics simulations, which further highlighted the residues involved in stabilizing the complex, yielding backbone RMSD values and per-residue interaction energy profiles that closely matched those obtained from the reference MD simulations for the retrospective cases. These results underscore the utility of this protocol for generating a plausible binding mode and investigating the energetic contributions of these regions to both the formation and the stabilization of the complexes.

The present work highlights the crucial strategy of guiding positively charged residues (arginines and lysines) toward negatively charged RNA grooves during SuMD supervision in investigating RNA-IDR interactions. This approach proved highly effective in capturing the multistep binding pathways and could serve as a general guideline for similar systems, particularly when structural or biochemical evidence indicates a dominant role for electrostatic steering. These results suggest the potential for broader applications of SuMD in studying RNA recognition by disordered regions.

The presented method still has some limitations: the definition of supervision coordinates remains dependent on prior biochemical or structural knowledge. Therefore, careful integration with experimental evidence is essential. Given the experimental challenges associated with resolving RNA-IDR complexes, especially those characterized by high flexibility and transient interactions, SuMD may serve as a complementary computational tool capable of bridging residue-level experimental data and three-dimensional structural hypotheses. The prospective SERF2-rG4 application illustrates how the method can support experimental findings by providing a structurally coherent model consistent with experimentally derived interaction patterns, even in the absence of a solved complex structure.

IDRs, due to their “degenerate specificity”, can interact with a wide range of nucleic acid targets and form RNA-IDP complexes implicated in diseases involving splicing dysregulation, RNA granule misregulation, and neurodegenerative conditions linked to RGG-rich proteins such as FUS and TAF15. Given the experimental challenges of probing these relevant complexes, SuMD could be a reliable tool that enables the computationally efficient investigation of these interactions with simulation times comparable to those achieved in protein-small molecule studies.

Furthermore, integration with complementary enhanced-sampling techniques, such as Thermal Titration MD (TTMD) (83), may enable systematic exploration of binding thermodynamics, stability, and energetic contributions of transient contacts.

The identification of key residues and binding motifs represents an essential step in guiding the rational design of small-molecule- or oligonucleotide-based inhibitors. In this scenario, SuMD serves as a valuable tool within structure-based drug discovery pipelines that target RNA–IDR interactions.

## Supplementary Material









## Data Availability

The code to perform SuMD simulations is available free of charge at https://github.com/molecularmodelingsection/SuMD. The script utilized to perform analysis on SuMD trajectories is also available at https://github.com/molecularmodelingsection/SuMD-analyzer. All trajectories presented in the article can be found at 10.5281/zenodo.17776719.

## Notes

All figures marked as supporting figures (Figures S1–S9) and tables (Table S1–S3) (PDF)
(VideoS1.mp4) SuMD simulation of the U1 snRNA stem-loop 4/SF3A1 ubiquitin-like domain (PDB ID: 7P0V); five panels: (top panels) molecular dynamics trajectory; (lower panels) time-dependent RMSD relative to the reference structure and the corresponding nucleic acid/protein interaction energy analyses (MP4)
(VideoS2.mp4) SuMD simulation of the U1 snRNA stem-loop 3/FUS RNA recognition motif (PDB ID: 6SNJ); six panels: (three upper panels) molecular dynamics trajectory; (lower panels) time-dependent RMSD and associated energetic analyses (MP4)
(VideoS3.mp4) SuMD simulation of the TERRA12 G-quadruplex/SERF2 complex; four panels: (upper panels) molecular dynamics trajectory (global view and nucleic acid-centered view); (lower panels) time-dependent per-residue interaction energy analyses for the nucleic acid and the protein (MP4)

## Abbreviations

GPU: graphic processing unit
PDB: Protein Data Bank
SuMD: supervised molecular dynamics
TTMD: thermal titration molecular dynamics
IDP/R: intrinsically disordered proteins/regions
