# Supplementary material for: Structuring Disorder via Supervised Molecular Dynamics: Uncovering Arginine-Glycine-Glycine-Mediated Ribonucleic Acid-Intrinsically Disordered Region Recognition Mechanisms
Source: J Chem Inf Model. 2026 May 7;66(10):6027–44. doi: 10.1021/acs.jcim.5c03135 (PMC13213911; doi:10.1021/acs.jcim.5c03135)
Supplement: Supplementary file 1 [file ci5c03135_si_001.pdf]

Supporting Information

Structuring Disorder via Supervised Molecular Dynamics (SuMD): Uncovering RGG-Mediated RNA-IDR Recognition Mechanisms

*Gianluca Novello, Andrea Dodaro, Silvia Menin, Chiara Cavastracci Strascia, Mattia Sturlese, Veronica Salmaso and Stefano Moro**

Molecular Modeling Section (MMS), Department of Pharmaceutical and Pharmacological Sciences, University of Padova, via Marzolo 5, 35131 Padova, Italy

Molecular Modeling Section (MMS), Department of Pharmaceutical and Pharmacological Sciences, University of Padova, via Marzolo 5, 35131 Padova, Italy

Corresponding Author: Stefano Moro - Molecular Modeling Section (MMS), Department of Pharmaceutical and Pharmacological Sciences, University of Padova, via Marzolo 5, 35131 Padova, Italy; https://orcid.org/0000-0002-7514-3802; Email: stefano.moro@unipd.it

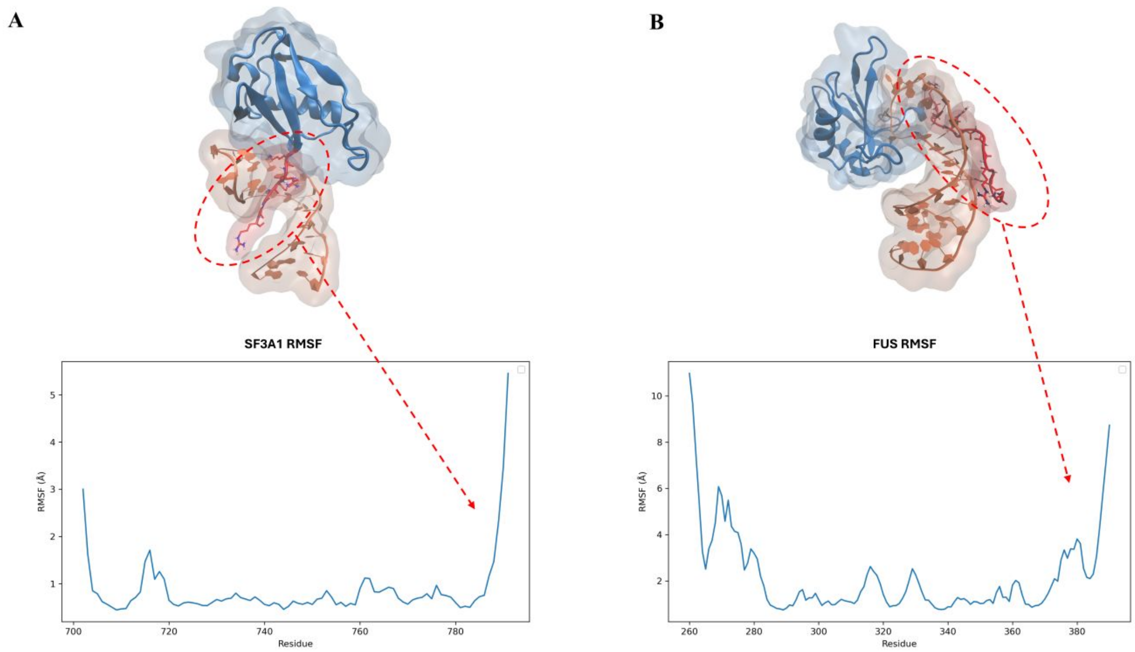

Figure S1. Comparative RMSF analysis of protein Cα atoms calculated over 500 ns classical MD simulations of the reference PDB complexes. Panel A: SF3A1-U1 SL4. Panel B: FUS-U1 SL3. In both complexes, the disordered RGG-containing tail is highlighted on the protein structure by a red dashed circle. The corresponding residues are indicated in the RMSF plots by arrows, allowing direct correlation between structural localization and flexibility profile.

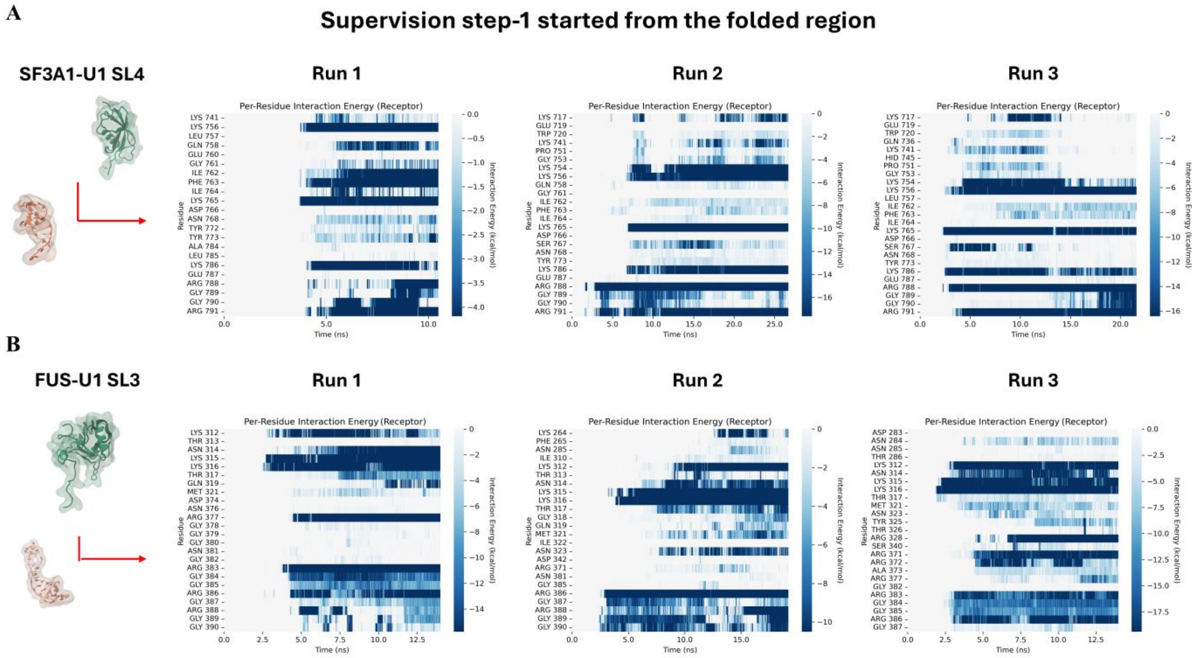

Figure S2. Per-residue protein-RNA interaction energy profiles obtained from triplicate SuMD simulations in which Supervision Step-1 was initially applied to residues located in the folded region of the protein. The analysis is shown for the SF3A1-U1 SL4 system (panel A) and the

FUS-U1 SL3 system (panel B). For each system, three independent replicas (Run 1-3) are reported.

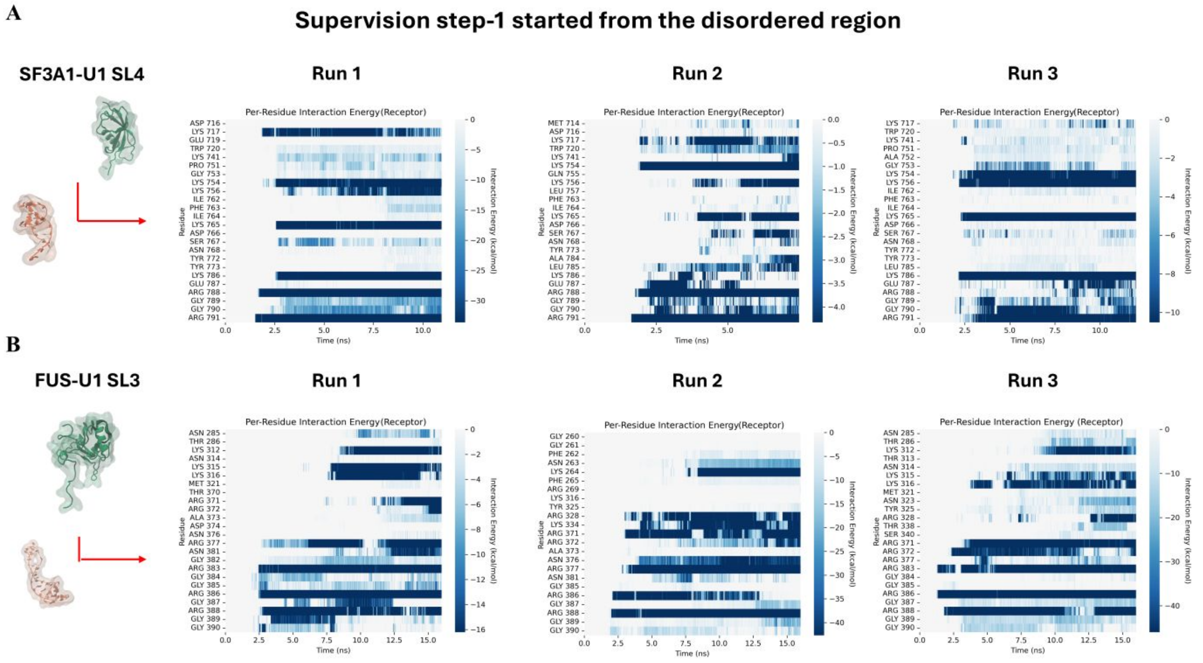

Figure S3. Per-residue protein-RNA interaction energy profiles obtained from triplicate SuMD simulations in which Supervision Step-1 was initially applied to residues located in the disordered region of the protein. The analysis is shown for the SF3A1-U1 SL4 system (panel A) and the FUS-U1 SL3 system (panel B). For each system, three independent replicas (Run 1-3) are reported.

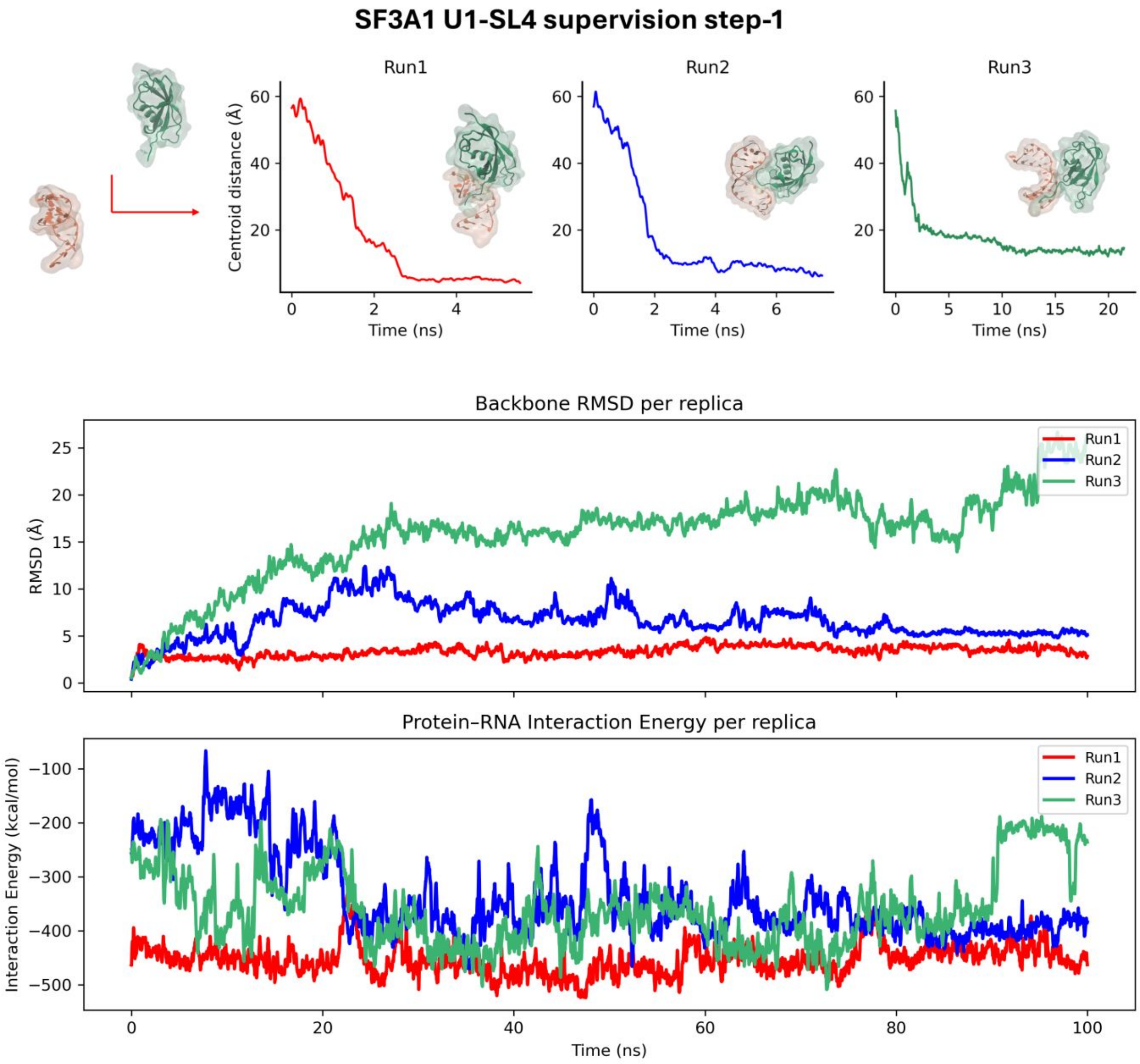

Figure S4. Supervision step-1 of the SF3A1-U1 SL4 complex. Top panels: time evolution of the supervised center-of-mass distance between protein and RNA for the three independent SuMD replicas (Run1, Run2, Run3). For each run, the last frame obtained at the end of the supervision step is shown alongside the corresponding distance profile (green for protein, orange for RNA). Middle panel: backbone RMSD over 100 ns of classical MD performed starting from the final supervised frame of each replica, reported separately for Run1, Run2, and Run3 (For this analysis, the RMSD was calculated with respect to the last frame obtained at the end of the supervision step, considering only the disordered tail residues). Bottom panel: protein-RNA interaction energy calculated over the same 100 ns MD trajectories for each replica.

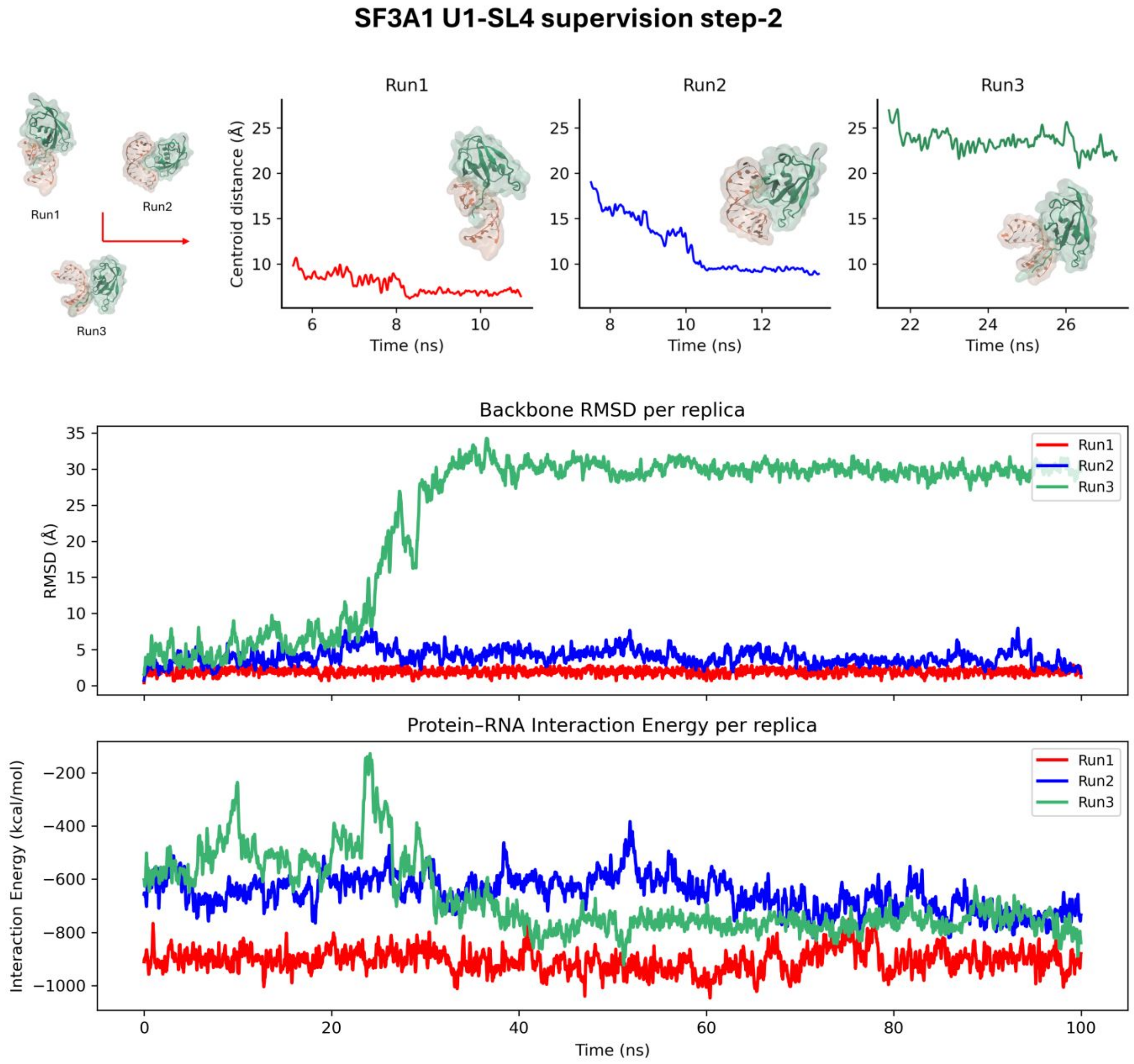

Figure S5. Supervision step-2 of the SF3A1-U1 SL4 complex. Top panels: time evolution of the supervised center-of-mass distance between protein and RNA for the three independent SuMD replicas (Run1, Run2, Run3). For each run, the last frame obtained at the end of the supervision step is shown alongside the corresponding distance profile (green for protein, orange for RNA). Middle panel: backbone RMSD over 100 ns of classical MD performed starting from the final supervised frame of each replica, reported separately for Run1, Run2, and Run3 (For this analysis, the RMSD was calculated with respect to the last frame obtained at the end of the supervision step, considering all the protein residues). Bottom panel: protein-RNA interaction energy calculated over the same 100 ns MD trajectories for each replica.

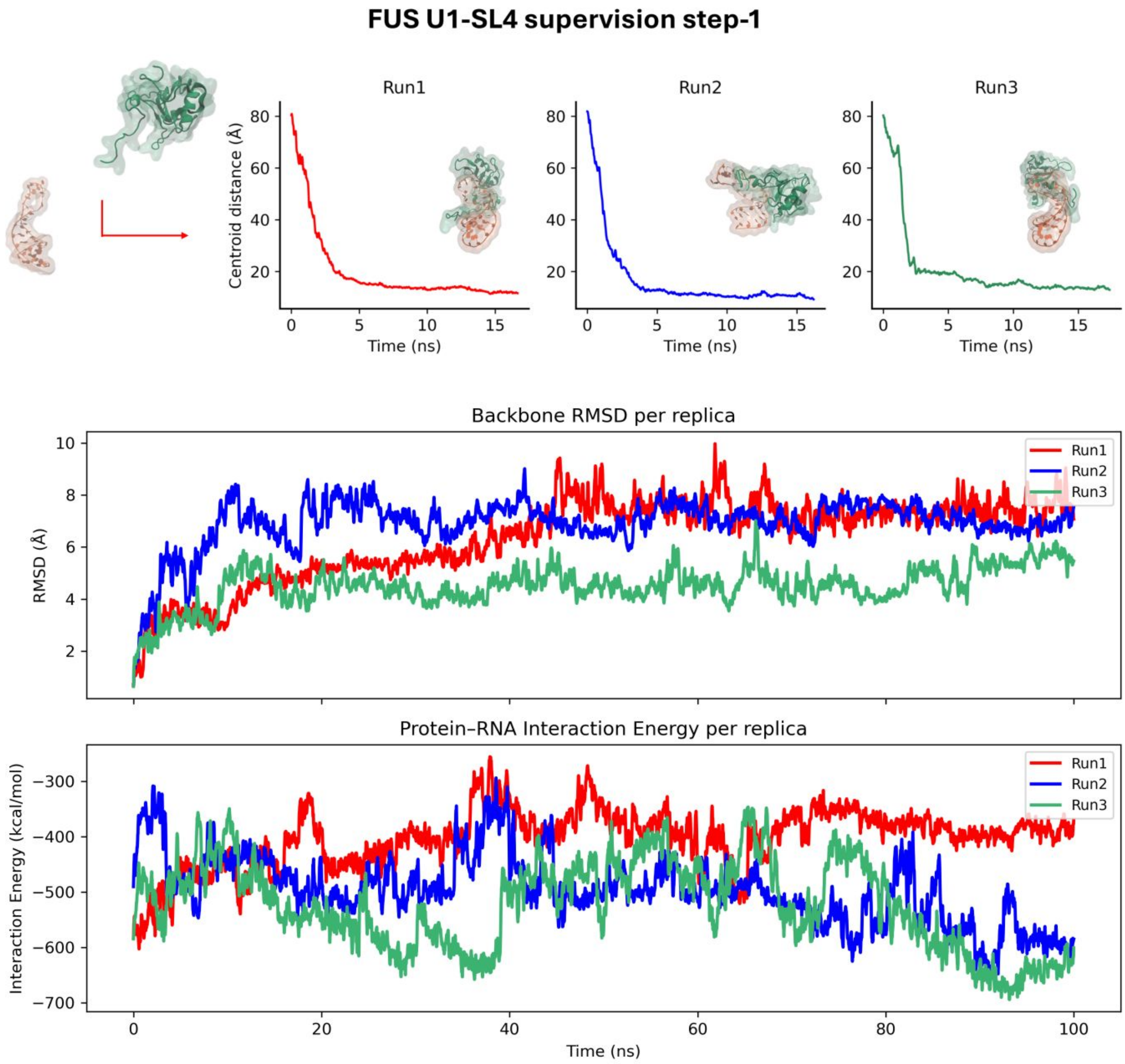

Figure S6. Supervision step-1 of the FUS-U1 SL3 complex. Top panels: time evolution of the supervised center-of-mass distance between protein and RNA for the three independent SuMD replicas (Run1, Run2, Run3). For each run, the last frame obtained at the end of the supervision step is shown alongside the corresponding distance profile (green for protein, orange for RNA). Middle panel: backbone RMSD over 100 ns of classical MD performed starting from the final supervised frame of each replica, reported separately for Run1, Run2, and Run3 (For this analysis, the RMSD was calculated with respect to the last frame obtained at the end of the supervision step, considering only the disordered tail residues). Bottom panel: protein-RNA interaction energy calculated over the same 100 ns MD trajectories for each replica.

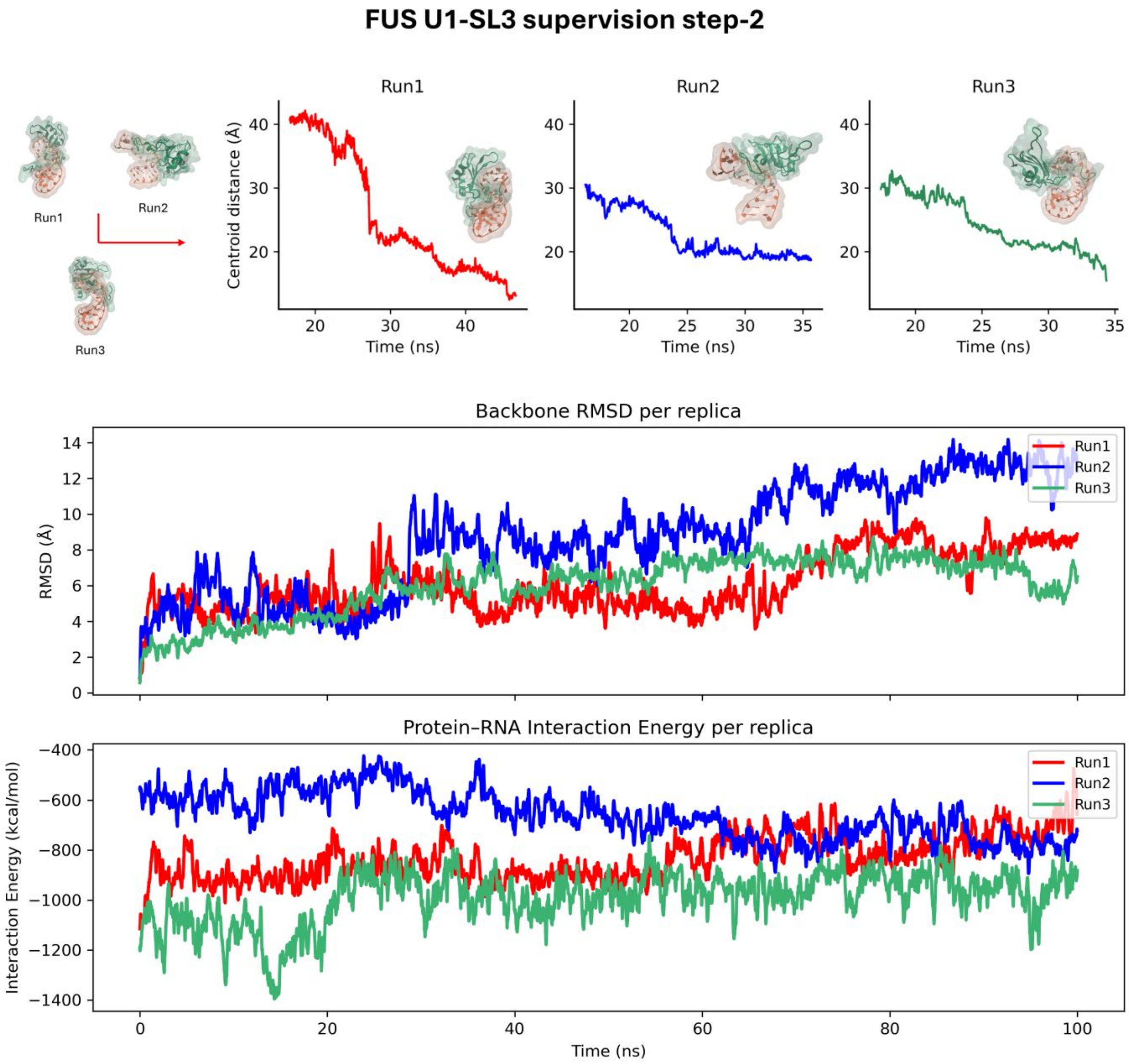

Figure S7. Supervision step-2 of the FUS-U1 SL3 complex. Top panels: time evolution of the supervised center-of-mass distance between protein and RNA for the three independent SuMD replicas (Run1, Run2, Run3). For each run, the last frame obtained at the end of the supervision step is shown alongside the corresponding distance profile (green for protein, orange for RNA). Middle panel: backbone RMSD over 100 ns of classical MD performed starting from the final supervised frame of each replica, reported separately for Run1, Run2, and Run3 (For this analysis, the RMSD was calculated with respect to the last frame obtained at the end of the supervision step, considering all the protein residues). Bottom panel: protein-RNA interaction energy calculated over the same 100 ns MD trajectories for each replica.

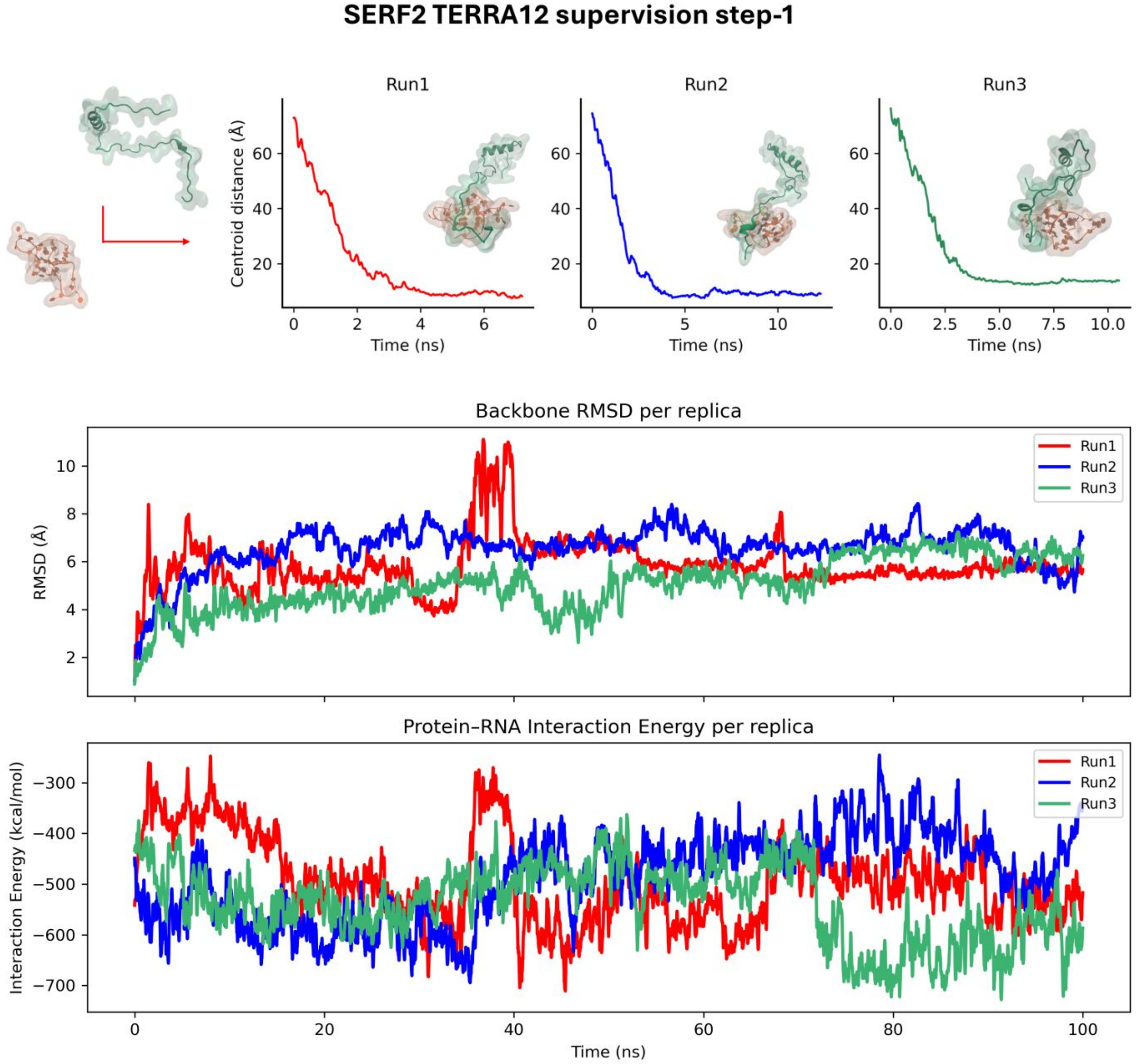

Figure S8. Supervision step-1 of the SERF2-TERRA12 complex. Top panels: time evolution of the supervised center-of-mass distance between protein and RNA for the three independent SuMD replicas (Run1, Run2, Run3). For each run, the last frame obtained at the end of the supervision step is shown alongside the corresponding distance profile (green for protein, orange for RNA). Middle panel: backbone RMSD over 100 ns of classical MD performed starting from the final supervised frame of each replica, reported separately for Run1, Run2, and Run3 (For this analysis, the RMSD was calculated with respect to the last frame obtained at the end of the supervision step, considering only the disordered tail residues). Bottom panel: protein-RNA interaction energy calculated over the same 100 ns MD trajectories for each replica.

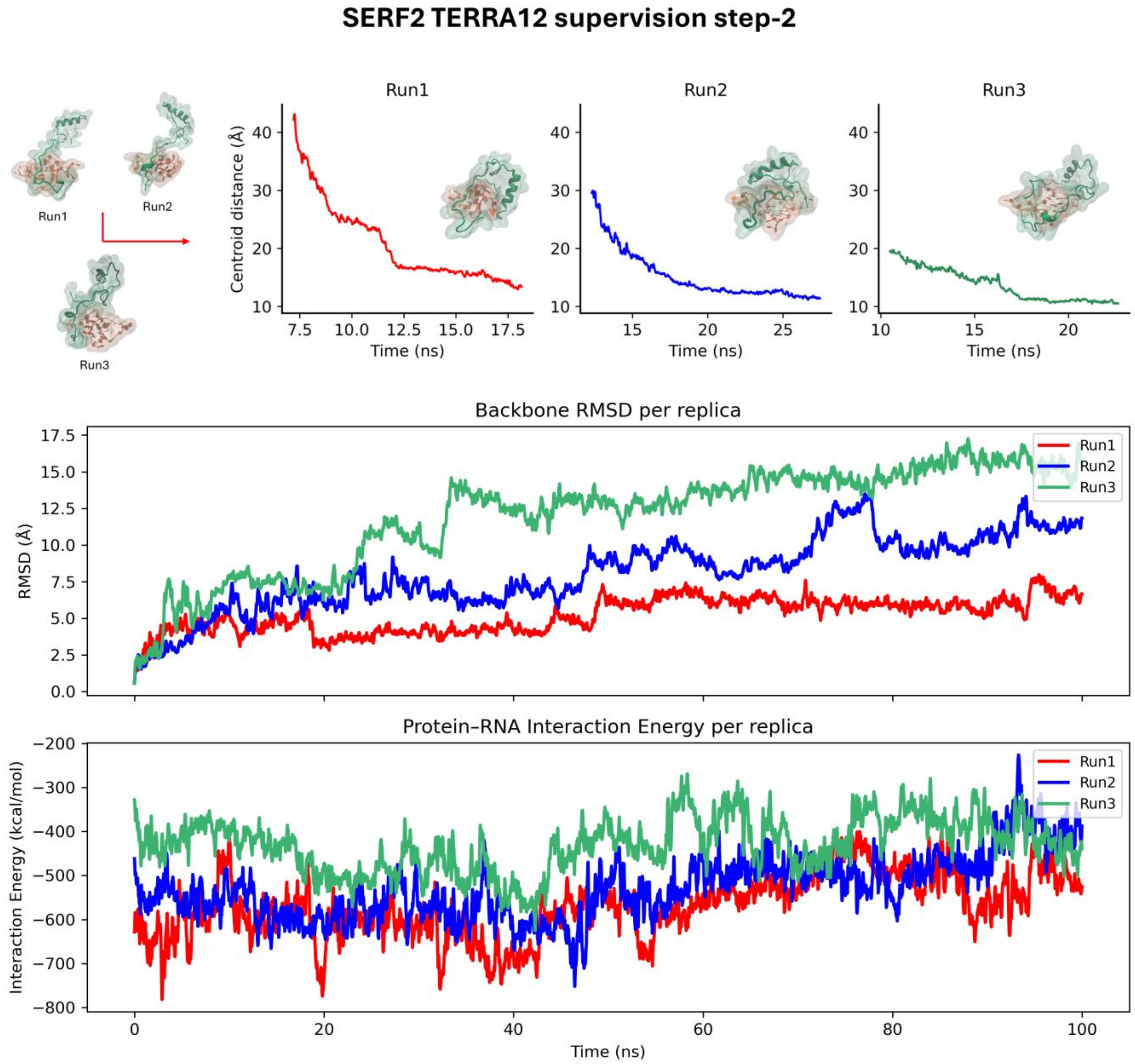

Figure S9. Supervision step-2 of the SERF2-TERRA12 complex. Top panels: time evolution of the supervised center-of-mass distance between protein and RNA for the three independent SuMD replicas (Run1, Run2, Run3). For each run, the last frame obtained at the end of the supervision step is shown alongside the corresponding distance profile (green for protein, orange for RNA). Middle panel: backbone RMSD over 100 ns of classical MD performed starting from the final supervised frame of each replica, reported separately for Run1, Run2, and Run3 (For this analysis, the RMSD was calculated with respect to the last frame obtained at the end of the supervision step, considering all the protein residues). Bottom panel: protein-RNA interaction energy calculated over the same 100 ns MD trajectories for each replica.

Case Study	Molecule	PDB ID	Residue Range	Sequence
SF3A1-U1 SL4	Protein (SF3A1 UBL domain)	7P0V	702-791	KGPVSIKVQVPNMQD KTEWKLNGQVLVFTL PLTDQVSVIKVKIHEA TGMPAGKQKLQYEGI FIKDSNSLAYYNMAN GAVIHLALKERGGR
SF3A1-U1 SL4	RNA (U1 snRNA Stem-Loop 4)	7P0V	139-162	GGGGACUGCGUUCG CGCUUUCCCC
FUS-U1 SL3	Protein (FUS RRM + RGG region)	6SNJ	260-390	GGFNKFGGPRDQGS RHDSEQDNSDNNTIF VQGLGENVTIESVAD YFKQIGIIKTNKKTGQ PMINLYTDRETGKLK GEATVSFDDPPSAKA AIDWFDGKEFSGNPIK VSFATRRADFNRGGG NGRGGRGRGG
FUS-U1 SL3	RNA (U1 snRNA Stem-Loop 3)	6SNJ	92-118	GGGAUUUCCCCAAA UGUGGGAAACUCCC
SERF2-TERRA12	Protein (SERF2)	9DT0	1-59	MTRGNQRELARQKN MKKQSDSVKGKRRD DGLSAAARKQRDSEI MQQKQKKANEKKEE PK
SERF2-TERRA12	RNA (TERRA12 G-quadruplex)	2KBP	1-24	UAGGGUUAGGGUUA GGGUUAGGGU

Table S1. Protein and RNA sequences employed in the three case studies investigated using the SuMD protocol. Residue numbering corresponds to the experimentally resolved constructs used in the simulations. Protein sequences are reported from N- to C-terminus, while RNA sequences are shown in the 5′→3′ direction.

Supervision strategy: Step-1 supervision on folded region		
System	Run	RMSD (of the supervised region) vs reference (Å)
SF3A1	Run 1	39.16
	Run 2	18.06

	Run 3	22.70
FUS	Run 1	64.81
	Run 2	21.68
	Run 3	62.33
Supervision strategy: Step-1 supervision on disordered region		
System	Run	RMSD (of the supervised region) vs reference (Å)
SF3A1	Run 1	3.41
	Run 2	10.92
	Run 3	20.08
FUS	Run 1	23.32
	Run 2	20.57
	Run 3	9.78
Supervision strategy: Step-2 supervision on folded region		
System	Run	RMSD (of the entire protein) vs reference (Å)
SF3A1	Run 1	4.20
	Run 2	33.64
	Run 3	27.61
FUS	Run 1	38.25
	Run 2	14.31
	Run 3	12.41

Table S2. RMSD values calculated relative to the experimental reference structures deposited in the PDB, obtained during preliminary tests aimed at evaluating alternative supervision strategies (folded or disordered) in SuMD simulations during Step-1 and 2.

System	Run	Step-1 protein backbone RMSD (Å)	Step-1 Interaction Energy (kcal/mol)	Step-2 protein backbone RMSD (Å)	Step-2 Interaction Energy (kcal/mol)
SF3A1-U1 SL4	Run 1	3.35 ± 0.61	-452.9 ± 31.4	1.93 ± 0.89	-904.5 ± 52.2
	Run 2	6.61 ± 1.87	-336.9 ± 80.0	3.98 ± 1.22	-651.3 ± 71.3
	Run 3	15.78 ± 4.69	-358.5 ± 71.8	23.57 ± 10.42	-677.1 ± 138.0

FUS-U1 SL3	Run 1	7.30 ± 0.80	-380.00 ± 40.00	6.09 ± 1.69	-835.20 ± 91.10
	Run 2	7.20 ± 0.60	-520.00 ± 60.00	8.69 ± 3.04	-660.80 ± 98.60
	Run 3	4.80 ± 0.60	-590.00 ± 70.00	6.00 ± 1.60	-992.10 ± 110.30
SERF2-TERRA12	Run 1	5.86 ± 1.11	-494.80 ± 86.00	5.20 ± 1.18	-569.60 ± 74.30
	Run 2	6.55 ± 0.93	-489.80 ± 88.70	8.09 ± 2.49	-528.50 ± 76.50
	Run 3	5.07 ± 1.11	-538.20 ± 77.40	11.96 ± 3.53	-430.20 ± 64.00

Table S3. Structural and energetic metrics for the three independent SuMD replicas performed for each investigated system. For each run, the table reports the average backbone RMSD and protein-RNA interaction energy calculated at the end of Supervision Step-1 and Step-2. Values are reported as mean ± standard deviation.
